# Boolean ErbB network reconstructions and perturbation simulations reveal individual drug response in different breast cancer cell lines

**DOI:** 10.1186/1752-0509-8-75

**Published:** 2014-06-25

**Authors:** Silvia Von der Heyde, Christian Bender, Frauke Henjes, Johanna Sonntag, Ulrike Korf, Tim Beißbarth

**Affiliations:** 1 Statistical Bioinformatics, Department of Medical Statistics, University Medical Center Göttingen, Humboldtallee 32, 37073 Göttingen, Germany; 2 TRON - Translational Oncology at the University Medical Center Mainz, Langenbeckstraße 1, 55131 Mainz, Germany; 3 Science for Life Laboratory, School of Biotechnology, KTH - Royal Institute of Technology, Box 1031, 17121 Solna, Sweden; 4 Division of Molecular Genome Analysis, German Cancer Research Center (DKFZ), Im Neuenheimer Feld 580, 69120, Heidelberg, Germany

**Keywords:** ErbB, RPPA, Network reconstruction, Boolean model, Breast cancer cell line, Drug resistance

## Abstract

**Background:**

Despite promising progress in targeted breast cancer therapy, drug resistance remains challenging. The monoclonal antibody drugs trastuzumab and pertuzumab as well as the small molecule inhibitor erlotinib were designed to prevent ErbB-2 and ErbB-1 receptor induced deregulated protein signalling, contributing to tumour progression. The oncogenic potential of ErbB receptors unfolds in case of overexpression or mutations. Dimerisation with other receptors allows to bypass pathway blockades. Our intention is to reconstruct the ErbB network to reveal resistance mechanisms. We used longitudinal proteomic data of ErbB receptors and downstream targets in the ErbB-2 amplified breast cancer cell lines BT474, SKBR3 and HCC1954 treated with erlotinib, trastuzumab or pertuzumab, alone or combined, up to 60 minutes and 30 hours, respectively. In a Boolean modelling approach, signalling networks were reconstructed based on these data in a cell line and time course specific manner, including prior literature knowledge. Finally, we simulated network response to inhibitor combinations to detect signalling nodes reflecting growth inhibition.

**Results:**

The networks pointed to cell line specific activation patterns of the MAPK and PI3K pathway. In BT474, the PI3K signal route was favoured, while in SKBR3, novel edges highlighted MAPK signalling. In HCC1954, the inferred edges stimulated both pathways. For example, we uncovered feedback loops amplifying PI3K signalling, in line with the known trastuzumab resistance of this cell line. In the perturbation simulations on the short-term networks, we analysed ERK1/2, AKT and p70S6K. The results indicated a pathway specific drug response, driven by the type of growth factor stimulus. HCC1954 revealed an edgetic type of PIK3CA-mutation, contributing to trastuzumab inefficacy. Drug impact on the AKT and ERK1/2 signalling axes is mirrored by effects on RB and RPS6, relating to phenotypic events like cell growth or proliferation. Therefore, we additionally analysed RB and RPS6 in the long-term networks.

**Conclusions:**

We derived protein interaction models for three breast cancer cell lines. Changes compared to the common reference network hint towards individual characteristics and potential drug resistance mechanisms. Simulation of perturbations were consistent with the experimental data, confirming our combined reverse and forward engineering approach as valuable for drug discovery and personalised medicine.

## Background

Longitudinal time course data are the basis for modelling signalling networks in a holistic systems biology approach in order to uncover mechanisms of signal transduction dynamics [1,2]. Network models provide novel insight [3,4] and allow us to perform efficiently simulations to predict systems behaviour or evaluate certain hypotheses [5]. Furthermore, combining perturbation experiments with the measurements of system dynamics seems to be even more efficient than time series data on their own [6-8]. Knock-outs or stimuli as directed perturbations support the systematic identification of regulatory relationships.

Quantitative models, based on differential equations, require explicit knowledge on the kinetics of the system of interest [9-12]. In contrast, the qualitative Boolean abstraction considers the components’ states as binary variables, being either active (1) or passive (0), but nevertheless encompasses the essential functionality [13,14]. Wang et al. stressed, that Boolean models have already been successfully applied in reverse engineering of proteomic signalling networks, and their reduced complexity is considered to be especially advantageous for large-scale systems [15]. To avoid the drawbacks of purely data- or literature-driven algorithms regarding completeness, generalisation or interpretability, combined approaches become more and more prominent in the area of network reconstruction [6,16,17]. Some reverse engineering approaches, like *ddepn*[6] or *CellNOptR*[18], ideally join perturbed time course input data and literature prior knowledge in network reconstruction, while preserving the simplicity of Boolean logic at the same time. Forward engineering methods allow subsequent analysis of the stable states of the reconstructed system. Hence, this may allow to deduce possible long-term behaviour of components activity under perturbations. Such approaches are integrated and freely available in the open source Python software package *BooleanNet*[19] or in the *R*[20] package *BoolNet*[21], for example. As reviewed by Samaga and Klampt [22], several software tools can be applied for the dynamic modeling of logical signal transduction networks. Among others, they exemplarily mentioned *GINsim*[23], *SQUAD*[24], *BooleanNet*[19], *ChemChains*[25], *Odefy*[26], and *BoolNet*[21].

Here we focus on protein signalling networks in breast cancer, representing the most common cancer type among women [27]. Breast cancer, as a heterogeneous disease, can be divided into subgroups, which differ in cellular properties as well as in prognosis. This requires individual therapy approaches, which are in the focus of current research and have partially already been realised.

Here we are interested in the ‘HER2-positive’ subtype of breast cancer, overexpressing the human epidermal growth factor receptor 2 (HER2, also termed ErbB-2). ErbB-2 is a receptor tyrosine kinase (RTK) and member of the epidermal growth factor (EGF) receptor family, consisting of three further RTKs, namely ErbB-1, ErbB-3 and ErbB-4. These receptors cooperatively function as homo- or heterodimers after activation via growth factors like EGF for ErbB-1 or heregulin (HRG) for ErbB-3 [28]. This initialises signalling cascades, pathologically contributing to tumourigenesis and tumour progression. Interestingly, different dimer formations induce different signalling pathways, like PI3K and MAPK, also with differing signalling strengths [29]. The role of the orphan receptor ErbB-2 in dysregulation of the ErbB network is of major interest, due to its overexpression in 10-20% of breast tumours, diagnosed as HER2-positive. Furthermore, its role as favoured dimerisation partner independent on ligand-activation implies oncogenic potential [30-32]. The therapeutic antibodies trastuzumab and pertuzumab have especially been designed to target ErbB-2 [33].

However, frequently occurring therapy resistance reduces the efficiency of targeted therapeutics [34-36]. This resistance is often associated with deregulated pathway activity [37,38] or bypasses via other RTKs, especially ErbB family members [39]. Mainly ErbB-1 expression has been anticipated as molecular cause to overcome impact of ErbB-2 targeting drugs. Small-molecule inhibitors such as erlotinib are already in use against non-small cell lung cancer [40] and pancreatic cancer [41].

Here we aim as a first step at the identification of individual drug response patterns and insights into drug resistance in HER2-positive breast cancer. ErbB-2 amplified cell lines were therefore subjected to short- and long-term drug treatment with erlotinib, pertuzumab and trastuzumab, alone or in combinations. Samples were analysed by reverse-phase protein arrays (RPPA) [42]. We were interested in synergistic benefits of combining erlotinib, pertuzumab or trastuzumab in ErbB-1 expressing, ErbB-2 amplified tumours with differing resistance phenotypes. Therefore three representative breast cancer cell lines were selected as model systems, namely BT474, SKBR3 and HCC1954, of which the latter is known to be trastuzumab resistant due to a PIK3CA mutation, while BT474 exhibits wild type behaviour [43]. The SKBR3 cell line is supposed to be pertuzumab resistant [44].

ErbB dimers predominantly activate the MAPK and PI3K pathway [29]. Therefore, we concentrated on the involved key regulators in fast downstream signalling. Among those were ERK1/2 and AKT, and also p70S6K, which is upstream influenced by both of the signalling axes. Phosphorylation of RPS6 and RB was used as long-term indicator for proliferation, cell cycle or tumour progression [28]. Prior literature knowledge on ErbB signalling was used as input for protein network reconstruction per cell line via *ddepn*. Beyond that, we inferred combined therapies that target ErbB family members, customised to the topology of the different subtypes. *BoolNet* was applied to compute stable cycles of protein activity states, so-called attractors, incorporating all possible treatment combinations. This way, optimal drug treatment to deactivate oncogenic proteins was identified.

## Methods

### Data

Protein abundance and phosphorylation measurements in BT474, SKBR3 and HCC1954 cells were carried out as described by Henjes et al. [28]. In principle, the RPPA protein array technology works as follows. Minimal amounts (1 nl volume) of cell lysate are spotted along with a serial dilution of control samples on nitrocellulose-coated glass slides using a printing robot (Aushon 2470 arrayer). Samples are organised as ordered subarrays so that they are addressable during the data analysis procedure, and a single slide can accommodate one or more subarrays. Each subarray is analysed using a highly specific detection antibody to measure the abundance of a certain protein or its phosphorylation rate. For each spot, the ratio of bound detection antibody is visualised using secondary antibodies labelled with near infrared (NIR) fluorescent dyes. Slides are scanned using the Odyssey scanner (LiCor Biosciences). Spot intensities are determined using a microarray image analysis software (GenePix).

Apart from the quantitative character, another advantage of the technology is the handling of large sample sets which protein abundance can be detected simultaneously in a high throughput fashion. 20-200 identical slides can be produced in parallel in a single print run.

In order to normalise the data spot-wise for deviant total protein concentrations due to spotting variance, staining with Fast Green FCF dye was employed [42]. Therefore, one slide was stained with the dye to determine the total protein content of each lysate spot and corresponding signal intensity correction factors. The spots on the remaining slides were divided by these correction factors and afterwards multiplied by the median value to scale the data back to the native range.

The RPPA data used here include data presented in Henjes et al. [28]. Additionally, further targets have been measured and were used for network reconstruction. The complete data set has been submitted to the Gene Expression Omnibus (GEO) with accession number GSE50109.

#### Short-term measurements

In the short-term measurements, trastuzumab, pertuzumab and erlotinib were added to the cells in starvation medium one hour before stimulation with the growth factors EGF and HRG. All possible 24 combinations of drugs and stimuli were measured. Application of the stimuli was defined as time point zero in the measurements. The growth factors were chosen to activate explicitly the MAPK and PI3K pathway. Lysate preparation was performed at ten time points, namely after 0, 4, 8, 12, 16, 20, 30, 40, 50 and 60 minutes. The drug treatment experiments comprised three biological replicates, whereas the inhibitor-free experiments incorporated five biological replicates. The experiments for the SKBR3 cell line comprised only two biological replicates of HRG stimulated cells under the triple drug combination. Each biological replicate was spotted in triplicate on the RPPA slides. To obtain short-term signal intensities, eleven antibodies for specific phosphorylation sites were selected according to quality checks, including inspection of corresponding dilution series and comparison to signals arising from secondary antibodies only. The chosen target proteins and respective antibodies are listed in Additional file 1.

#### Long-term measurements

For long-term measurements, no explicit ligand stimulation was performed. Instead, cells were incubated in full growth medium for 24 hours prior to adding the three mentioned therapeutics in double combinations or as triplet. Single drug treatment was just conducted with erlotinib. Full growth medium was used to avoid confounding effects of nutrient deficiency. Protein abundance was also quantified without any drug application. The measuring points included 0, 1, 2, 4, 6, 8, 12, 18, 24 and 30 hours with three biological and technical replicates each. At time point 18, only two biological replicates were available. Additional file 1 displays the 21 targets of interest for long-term signalling.

### Statistical inference of drug effects

To determine, whether a specific drug treatment revealed an inhibiting effect on the signal intensities of the proteins, we applied the following method. Firstly, for each protein and (combinatorial) drug treatment we linearly modelled the signal intensities as depending on the factors *time* and *group*, i.e. no drug treatment versus drug treatment. If the interaction of both factors showed a significant (p-value < 0.05) influence on the signal intensity, we further applied a Wilcoxon rank sum test for the measurements at time point 60 minutes for the short-term data, or at time point 30 hours for the long-term data. Thereby, we tested for significantly (p-value < 0.05) smaller intensity values in the drug treated group. The drug treatments with a significant test result were considered as efficient inhibitors. The therapeutic (combination) with the smallest p-value was defined as the optimal one.

### Literature prior knowledge

We manually determined two reference networks, i.e. one for each time course, as initial joint hypotheses for all of the three breast cancer cell lines. Because emphasis was put on phosphoproteomic signalling, this was mainly based on PhosphoSitePlus^®;^[45]. Several publications confirm these assumptions, as depicted in Additional file 2.

### Network reconstruction

For Boolean network reconstruction, we chose the method of dynamic deterministic effects propagation networks (DDEPN) [6]. This method was particularly tailored to perturbed longitudinal protein phosphorylation data. It is based on the DEPN approach [46], which stands for deterministic effects propagation networks. The determinism is related to the way of perturbation effect propagation in the networks from parent to child nodes, implying transitively closed graphs. The dynamic version of Bender et al. [6,47] differs with respect to the integration of perturbed time course measurements. While the DEPN approach requires many perturbations, like knock-downs, but only few time points, which are regarded as independent measurements, *ddepn* is designed for longer time series without the necessity of many or all network nodes being perturbed. The latter situation, i.e. few perturbations by drug interventions, reflected the design of the RPPA experiments under consideration here, hence leading to the application of *ddepn*. Most network reconstruction algorithms have been designed for gene expression data from microarray measurements [7], which differ from (phospho-)protein data regarding the amount of involved network nodes. Many current methods are tailored to the inference of gene regulatory networks based on static measurements at one time point, reflecting the steady state of the system under consideration [48]. The longitudinal time course data used here require a suitable method, as provided by *ddepn*. The method of Bender et al. was shown to outperform two dynamical Bayesian network approaches, and to be capable of inferring known signalling cascades in the ErbB pathway [47]. A further advantage was the public availability of *ddepn* as an *R*[20] package.

The reconstruction procedure is depicted in Additional file 3, and the core elements are described according to [6,47] in the following. The protein interaction networks are modelled as directed, possibly cyclic, graphs, with nodes *V*={*v*_*i*_:*i*∈1,…,*N*} representing proteins and edges representing interactions. Also the external perturbations, i.e. the drugs and growth factors in our case, are modelled as nodes. The edge types can be either activating or inhibiting, denoted by 1 and -1, respectively, in the adjacency matrix *Φ*=*V*×*V*→{0,1,−1} of the network. An entry of zero indicates no edge between two nodes. So each edge incorporates a pair of nodes {*ϕ*_*i* *j*_:*i*,*j*∈1,…,*N*}. The measurement data, which form the basis for the reconstruction, are stored in a matrix *D*={*d*_*i* *t* *r*_:*i*∈1,…,*N*,*t*∈1,…,*T*,*r*∈1,…,*R*}, considering *T* time points and *R* replicates.

For the inference of a network structure, optimally fitting to the data, we applied the stochastic Markov Chain Monte Carlo (MCMC) approach of *ddepn*, called *inhibMCMC*, in which the space of possible networks is sampled, based on posterior probabilities. It extends a Metropolis-Hastings type of MCMC sampler by the capability of sampling two edge types directly, i.e. activation and inhibition. The posterior distribution of a network *Φ* given the data *D*, is defined as $P(\Phi |D)=\frac{P(D|\Phi )P(\Phi )}{P(D)}\propto P(D|\Phi )P(\Phi )$, with *P*(*Φ*) as the prior probability distribution and *P*(*D*|*Φ*) as the likelihood of the data given the network. The latter is defined in [47] as $p(D|\Phi )=p(D|{\hat{\Gamma }}^{*},\hat{\Theta })=\overset{T}{\underset{t=1}{\prod }}\overset{N}{\underset{i=1}{\prod }}\overset{R}{\underset{r=1}{\prod }}p(d_{\text{itr}}|{\hat{\theta }}_{i{\hat{\gamma }}_{\text{itr}}^{*}})$, where $\Gamma^{*}=\left\{\gamma_{\text{itr}}^{*}:i\in 1,\ldots ,N,t\in 1,\ldots ,T,r\in 1,\ldots ,R\right\}$ denotes the optimized system state matrix, containing active and passive states per protein and time point. It is estimated in the following way. Assuming that the proteins can be either active (1) or inactive (0), signalling dynamics are modelled by Boolean signal propagation for a given network. All nodes, except the permanently active perturbations, are therefore initialised with inactive states. The transition rule is that children nodes get activated if at least one activating parent node is active and all inhibiting ones are inactive. In this way, all reachable system states are computed and stored in a matrix *Γ*={*γ*_*i* *k*_∈{0,1}:*i*∈1,…,*N*,*k*∈1,…,*M*}, holding column-wise the activation states of all proteins at transition step *k*. The amount of transitions is limited by 0<*M*≤2^*N*^. This state matrix has to be optimized, as it is not related to the measured time points yet. The true unknown state sequence over time is represented by *Γ*^∗^, which is estimated by a hidden Markov model (HMM). The resulting ${\hat{\Gamma }}^{*}$ indicates whether a data point *d*_*i* *t* *r*_ has an underlying active (1) or passive (0) normal distribution

$$d_{\text{itr}}∼\left\{\begin{array}{cc} N(\mu_{i0},\sigma_{i0}), & \;\text{if}\;\;{\hat{\gamma }}_{\text{itr}}^{*}=0\\ N(\mu_{i1},\sigma_{i1}), & \;\text{if}\;\;{\hat{\gamma }}_{\text{itr}}^{*}=1. \end{array}\right)$$

The distribution parameters are for each protein estimated as empirical mean and standard deviation of all measurements for the considered protein in the corresponding class, yielding the parameter matrix $\hat{\Theta }=\left\{{\hat{\theta }}_{i0},{\hat{\theta }}_{i1}\right\}=\left\{\left({\hat{\mu }}_{i0},{\hat{\sigma }}_{i0}),({\hat{\mu }}_{i1},{\hat{\sigma }}_{i1}\right)\right\}\;\forall i\in 1,\ldots ,N$.

The prior probability distribution *P*(*Φ*) includes penalisation of differences between the network structure *Φ* and a user-defined prior belief *B*=*V*×*V*→[ −1,1], where the absolute value correlates with the confidence in an edge. Here we chose *B*=*V*×*V*→{0,1,−1}, assuming in advance specific activating, inhibiting or missing edges with maximum confidence. We made use of the Laplace prior model (*laplaceinhib*), accounting for both edge types, i.e. activation and inhibition. The prior belief for an edge is defined as $P(\phi_{\text{ij}}|b_{\text{ij}},\lambda ,\gamma )=\frac{1}{2\lambda }e^{\frac{-\Delta_{\text{ij}}}{\lambda }}$, including a weighted difference term *Δ*_*i* *j*_=|*ϕ*_*i* *j*_−*b*_*i* *j*_|^*γ*^ with a weight exponent $\gamma \in {\mathbb{R}}^{+}$. As the edge probabilities are assumed to be independent, the prior belief for a network structure *Φ* is derived as the product of those, i.e. $P(\Phi |B,\lambda ,\gamma )=\underset{i,j}{\prod }P(\phi_{\text{ij}}|b_{\text{ij}},\lambda ,\gamma ),\;i,j\in \left\{1,\ldots ,N\right\}$. The individual edge probabilities lie between 0 and $\frac{1}{2\lambda }\;\forall \lambda ,\gamma \in {\mathbb{R}}^{+}$. The protein interactions corresponding to our chosen prior are displayed in Additional file 2. The prior’s impact strength was emphasised in such a way, that only strongly deviating data influence the network structure, because the ErbB wiring as well as the MAPK and PI3K pathways are well examined in literature. This prioritisation is reflected in the hyperparameter *λ* set to 0.0001. For the parameter *γ* we chose one, neglecting extra penalisation of deviation from the prior. These settings should preserve robustness, but at the same time allow enough impact strength of strongly differing data values.

The network inference via *inhibMCMC* spanned 50,000 iterations with the first 25,000 iterative steps as burn-in phase. To ensure convergence, ten parallel MCMC chains were run, each initialised with a starting network. Convergence was validated via Gelman diagnostic [49]. Nine of the initial ten networks were randomly generated, i.e. for the defined nodes activating, inhibiting or no edges were sampled. The remaining network assumed no connections between the nodes. These initial networks were pruned to the following constraints. Firstly, the nodes related to the growth factors and drugs must not have any ingoing edges. Above that, the indegree of all nodes was limited to four. Finally, no self-loops were allowed. To find significantly occurring edges among the independent runs, merging into a consensus network, a Wilcoxon rank sum testing procedure was used. In detail, in each run the amount of sampled activations and inhibitions per edge was counted and divided by the total number of sampled edges. Subsequently the null-hypothesis was tested, whether the means of these ten edge-specific confidence values equal the same for activation and inhibition. In case of not rejecting the null-hypothesis, coming along with an adjusted p-value exceeding the significance level *α*=0.05, no edge was assumed. Otherwise, the respective alternative determined the type of interaction. Adjustment for multiple testing followed the method of Benjamini and Hochberg, controlling the false discovery rate [50]. The whole procedure was embedded into a leave-one-out cross-validation approach. So each of the ten MCMC chains was left out once, and the testing algorithm was applied to the remaining runs. An edge was included in the final consensus network if it occurred in all of the cross validation runs. Finally, to prevent excessive spurious or obsolete connections ascribable to transitivity, as argued by Bo Na Ki et al. [51], newly reconstructed edges were successively added to the prior network according to *ddepn* significance and fit of resulting attractor states to the observations of Henjes et al. [28].

### Perturbation simulations

To figure out which input of drug combination leads to a certain attractor state of the reconstructed network system, the *R* package *BoolNet*[21] was applied. The motivation was based on the assumption that attractors, representing cycles of states, comprise the stable states of cell function. In those states networks mostly reside. Hence, they mirror system phenotypes, dependent on the perturbation context. To the best of our knowledge, apart from *BoolNet*, there are hardly any *R* packages offering attractor calculations for Boolean networks. This package supports import of networks in form of files containing Boolean formulas. So it could be easily integrated in our workflow as subsequent analysis step after network reconstruction.

We used its functionality to identify attractors in a synchronous and an asynchronous way. The resulting attractors were steady-state attractors. These consist of only one state, in which all transitions from this state result. These attractors are identical for synchronous and asynchronous updates. We focused on the steady-states, as these should reflect the homoeostatic system state of the cell lines. Intermediate transition states would be interesting as well, but due to the large amount of the involved targets, it would have been too complex to analyse those here in detail.

The search started from predefined initial states of the network nodes. The drug and growth factor nodes were fixed to specific values, reflecting the conducted experiment to be simulated. For short-term signalling, perturbations included all possible combinations of the therapeutics under the combined stimulus of EGF and HRG. Although the data of separate stimulation with EGF and HRG was used for network reconstructions, here we focused on the combined treatment, representing a more natural tumour environment than a single growth factor alone. Two possible binary states, i.e. active (1) or passive (0), to the power of three different drugs led to eight possible combinations. These were used as fixed input conditions, as the effect was assumed to be continuously valid. Analogously, the growth factors were permanently fixed to one. The remaining protein activity start states were initialised with zero. These components were flexible towards updates. In the long-term measurements, no growth factors were involved but full growth medium. This was defined as one stimulating input *S*, initially activating the ErbB receptors. This also led to eight fixed input combinations.

*BoolNet* expects network representation in form of logical interaction rules as input. In contrast, *ddepn* delivers network reconstruction output in terms of adjacency matrices. Therefore, we incorporated an interface function into the *ddepn* package, called *adjacencyMatrix_to_logicalRules*. In detail, the *loadNetwork* function of *BoolNet* requires a file containing row-wise logical activation rules of each network node. Each row looks like ‘target node, (activator_1 | activator_2) & !(inhibitor_1 | inhibitor_2)’, here exemplary for a node with two ingoing activating and inhibiting edges each. The logical OR operator is encoded by ‘ |’, the logical AND is encoded by ‘&’, and logical negation is represented by ‘!’. Accordingly, all of the *A* inferred activating nodes *V*_+_={*v*_*a*_:*a*∈1,…,*A*, *A*<*N*} of a target node *v*_*j*_, represented by an adjacency matrix entry *ϕ*_*a* *j*_=1, and *v*_*j*_ itself were connected via OR operators. This ensured that at least one of the activators or the target protein itself had to be active to activate the target node. Analogously, the *I* inhibiting nodes *V*_−_={*v*_*i*_:*i*∈1,…,*I*, *I*<*N*} with *ϕ*_*i* *j*_=−1 were connected via OR operators. A logical negation operator was attached to ensure that the activity of one of the nodes *v*_*i*_ would result in an inactive node *v*_*j*_. Both sets of activators and negated inhibitors were then connected via a logical AND operator. After conversion of the adjacency matrices to logical rules, those were implemented in *BoolNet* into a computational model, to perform perturbation simulations per cell line and time course as well as subsequent analyses of the resulting attractor states.

## Results and discussion

**Figure 1:**
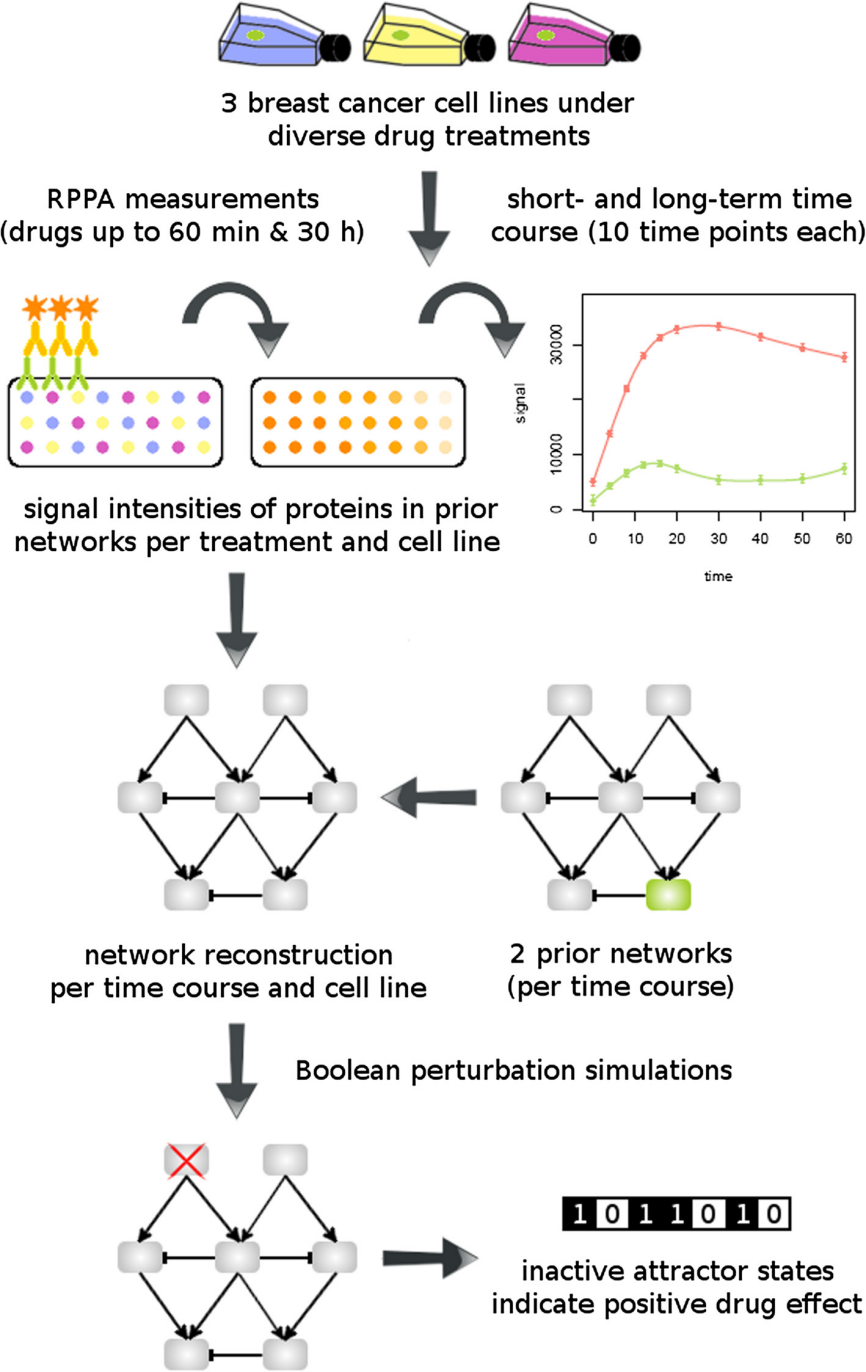
**Modelling workflow.** The figure summarises the applied modelling approach. RPPA data of three individual breast cancer cell lines were generated under short- and long-term drug treatment. They constituted the basis for network reconstruction in combination with prior literature knowledge about protein wiring. The reconstructed networks per cell line and time course in turn underwent Boolean perturbation simulations to reveal optimal drug treatments.

**Figure 2:**
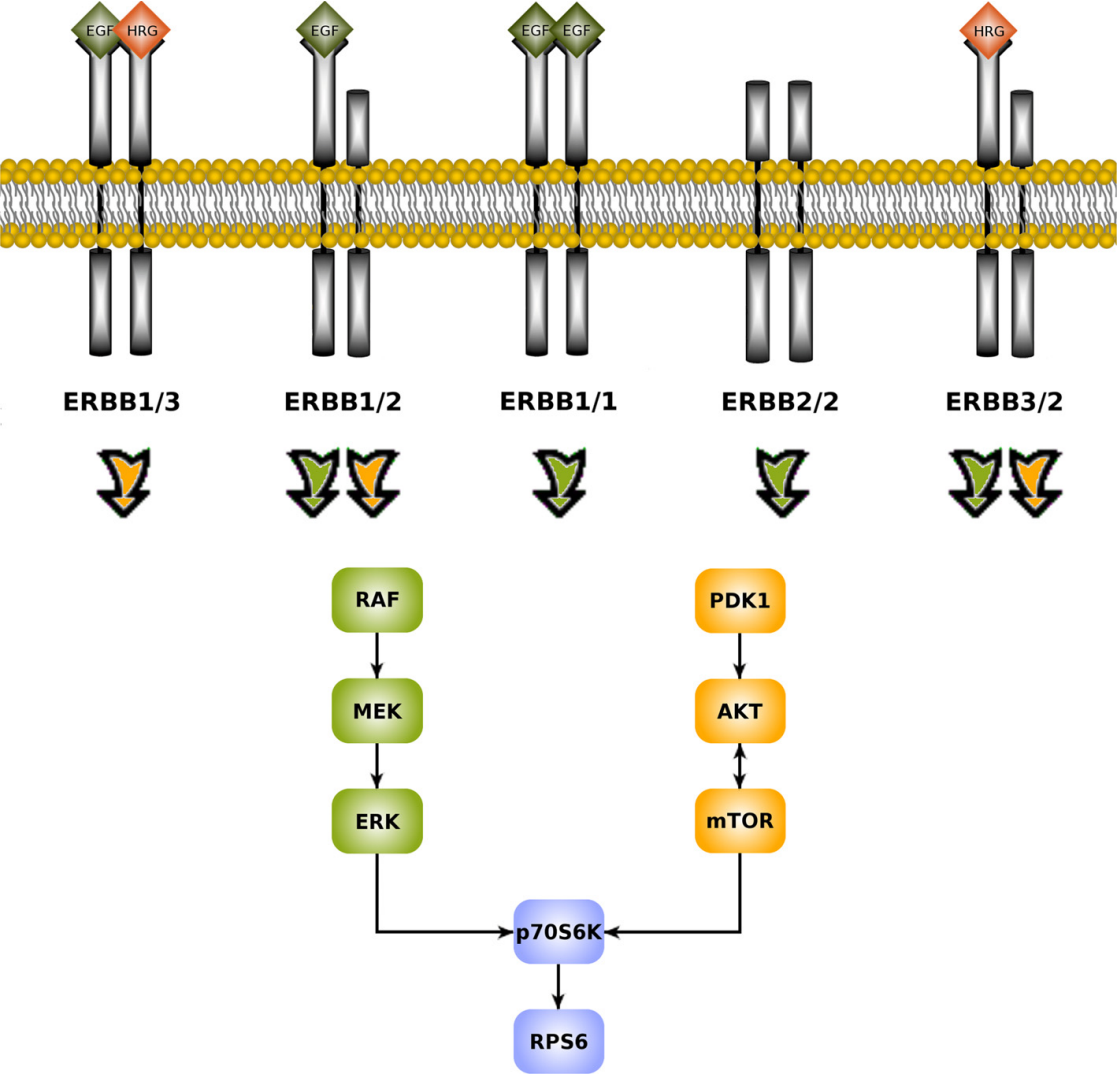
**Scheme of ErbB dimers related MAPK and PI3K pathway activation.** The figure depicts the different homo- and heterodimers of ErbB receptors, induced upon activation via the ligands EGF or HRG. The active dimers then initialise the MAPK and PI3K signalling cascades. The orange (PI3K) and green (MAPK) arrows denote, which dimer activates which pathway.

The complete workflow, holding for both, short- and long-term analysis, is depicted in Figure 1. For a better understanding of the discussion on MAPK and PI3K signalling, Figure 2 displays the interactions between the main MAPK and PI3K targets of the ErbB prior networks. It shows the preferred pathway activations by all possible homo- and heterodimers formed upon ligand binding to the ErbB-1 and ErbB-3 receptors [9,29,52-54]. The confidence values, representing the likeliness of the reconstructed network edges, are shown in Additional file 4.

### Short-term signalling network reconstruction

**Figure 3:**
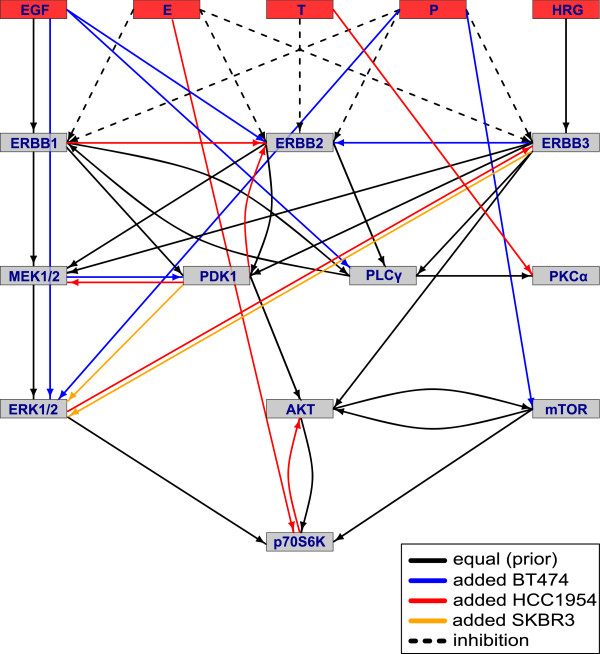
**Reconstructed short-term signalling networks.** The figure displays the reconstructed short-term signalling networks coloured according to the preserved prior reference network (black) and newly inferred (*added*) individual edges per cell line. Target proteins are represented as rectangles with stimuli and drugs coloured in red. The three drug names erlotinib, trastuzumab and pertuzumab are abbreviated via their first letters. Solid arrows denote activating interactions while dashed ones represent inhibitions.

The short-term signalling networks, reconstructed by the *ddepn* algorithm, are depicted in Figure 3. The equivalent Boolean logical interaction rules are listed in Additional file 5. In comparison to the prior network, newly inferred edges were specific for each cell line, and all of them were activating. For HCC1954 and BT474, seven additional edges were reconstructed, while in SKBR3 only two new edges were reconstructed. No prior edge deletion or type reversal took place.

#### HCC1954 is driven by the PI3K as well as the MAPK pathway

In HCC1954, the new edges contributed to both, PI3K and MAPK, signalling. The interaction ErbB-1 →ErbB-2 reflected a dominant role of heterodimerisation of both receptors, as described by Henjes et al. [28]. The fact that it was specifically inferred for HCC1954, pointed to hyperactive ErbB-1/2 heterodimers here. These are known to trigger the MAPK but also, to a lesser extent, the PI3K pathway. The link PDK1 →MEK1/2, supported by Sato et al. [55], stressed crosstalk between these pathways, placing PDK1 into a key position in the PI3K pathway, and MEK1/2 in the MAPK pathway, respectively. Two of the new edges in HCC1954, PDK1 →ErbB-2 and p70S6K →AKT, contributed to feedback loops, which were not present in the other two cell lines. Such a topological network element could stabilise the known trastuzumab resistance by boosting the oncogenic effect of ErbB-2 and the mutant hyperactive PI3K pathway. Evidence for the feedback mechanism involving PDK1 was provided by Maurer et al. [56] and Tseng et al. [57]. Vega et al. noted an indirect activation of AKT by p70S6K via mTOR [58].

#### BT474 is driven by the PI3K pathway, while SKBR3 is driven by the MAPK pathway

Comparably to HCC1954, in BT474 an edge indicating hyperactive heterodimers was found, namely ErbB-3 →ErbB-2, here interestingly with a strong impact on AKT [28]. BT474 is known to contain a rare type of PIK3CA mutation [43]. Pathway crosstalk was also observed in BT474, but here MEK1/2 activated PDK1, and not vice versa like in HCC1954. This edge was supported by Frödin et al. [59], underlining dominant PI3K signalling in this cell line.

The newly detected interactions in SKBR3 started from ErbB-3 and PDK1, and both activated ERK1/2. This reflected a dominant MAPK pathway, in which ErbB-3 →ERK1/2 was interpretable as indirect stimulation of ERK1/2 via MEK1/2, activated by ErbB-2/3 dimers [55].

### Perturbation simulations on short-term networks

Perturbations included all possible combinations of the therapeutics erlotinib, pertuzumab and trastuzumab under combined stimulation of EGF and HRG. All inferred attractors were simple and consisted of one steady-state. This means that all transitions from this state result in the state itself. Table 1 summarises all simulation outcomes for the attractors of the AKT and ERK1/2 proteins, as those are key players in the PI3K (AKT) and MAPK (ERK1/2) pathways. Additionally, the results for p70S6K are listed there, as both pathways regulate this protein [60].

**Table 1:** Attractor states of short-term perturbation simulations

	**BT474**						**HCC1954**						**SKBR3**					
**Simulation**	**AKT**		**ERK1/2**		**p70S6K**		**AKT**		**ERK1/2**		**p70S6K**		**AKT**		**ERK1/2**		**p70S6K**	
	**A**	**E**	**A**	**E**	**A**	**E**	**A**	**E**	**A**	**E**	**A**	**E**	**A**	**E**	**A**	**E**	**A**	**E**
X	**1**		**1**		**1**		**1**		**1**		**1**		**1**		**1**		**1**	
E	0	1	1	0	1	0	**1**		**0**		**1**		0	1	**0**		0	1
P	**1**		**1**		**1**		0	1	0	1	0	1	0	1	0	1	0	1
T	**1**		**1**		**1**		**1**		**1**		**1**		**1**		**1**		**1**	
E, P	**1**		1	0	1	0	1	0	**0**		1	0	**0**		**0**		0	1
E, T	0	1	1	0	**1**		**1**		0	1	**1**		0	1	**0**		0	1
P, T	**1**		**1**		**1**		0	1	**0**		0	1	**0**		**0**		0	1
E, P, T	**1**		1	0	1	0	1	0	**0**		**1**		**0**		**0**		**0**	

The therapeutics erlotinib, trastuzumab and pertuzumab, abbreviated by first letters, that were permanently active besides EGF and HRG in the simulated perturbation conditions are stored in the column *Simulation*. No simulated drug treatment is denoted by ‘X’. The *A* columns hold the attractor states of the proteins AKT, ERK1/2 and p70S6K, associated with the perturbations. The *E* columns contain the protein activity status, statistically deduced from the experimental data. In case of a significant (p-value < 0.05) combined influence of both, drug treatment and time, on the protein signal intensity, a Wilcoxon rank sum test was conducted for the measurements at time point 60 minutes. The drug treatments leading to significantly (p-value < 0.05) smaller intensity values compared to the control measurement ‘X’ were considered as efficient inhibitors, resulting in a table entry of zero. Consistency between simulations and experimental observations is printed in bold.

**Figure 4:**
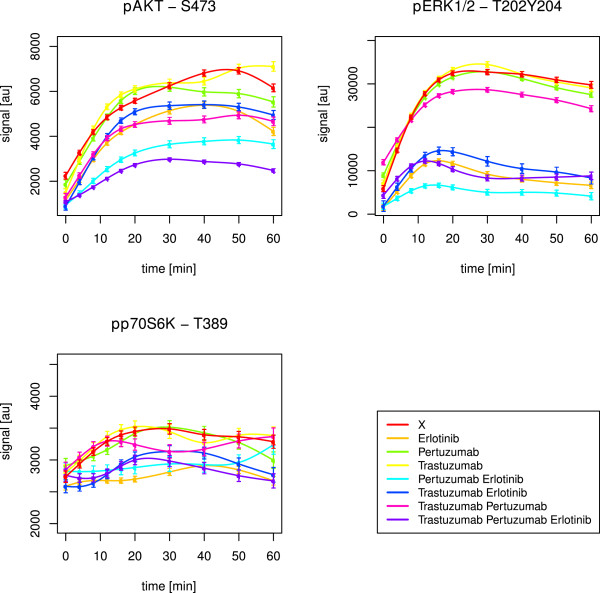
**SKBR3 short-term time courses of AKT, ERK1/2 and p70S6K.** The figure shows splines and related standard error bars of the measured RPPA data for AKT, ERK1/2 and p70S6K after combined EGF and HRG stimulation in the SKBR3 cell line. The measurements included ten time points up to 60 minutes. The different drug treatments are marked by different colours with ‘X’ denoting no drug treatment.

**Figure 5:**
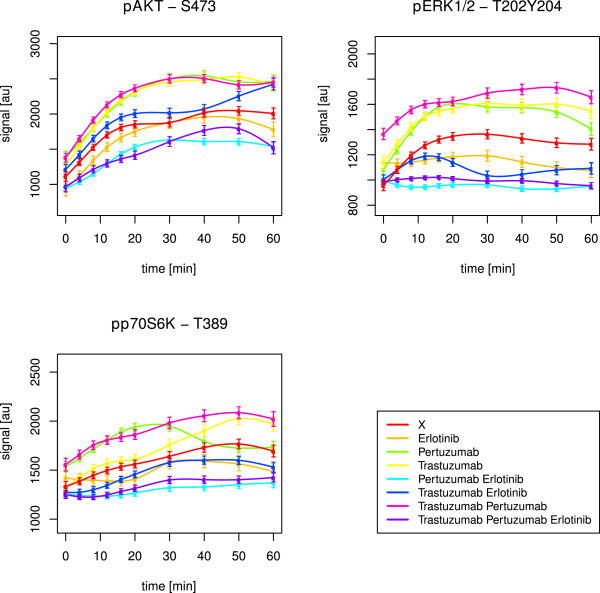
**BT474 short-term time courses of AKT, ERK1/2 and p70S6K.** The figure shows splines and related standard error bars of the measured RPPA data for AKT, ERK1/2 and p70S6K after combined EGF and HRG stimulation in the BT474 cell line. The measurements included ten time points up to 60 minutes. The different drug treatments are marked by different colours with ‘X’ denoting no drug treatment.

**Figure 6:**
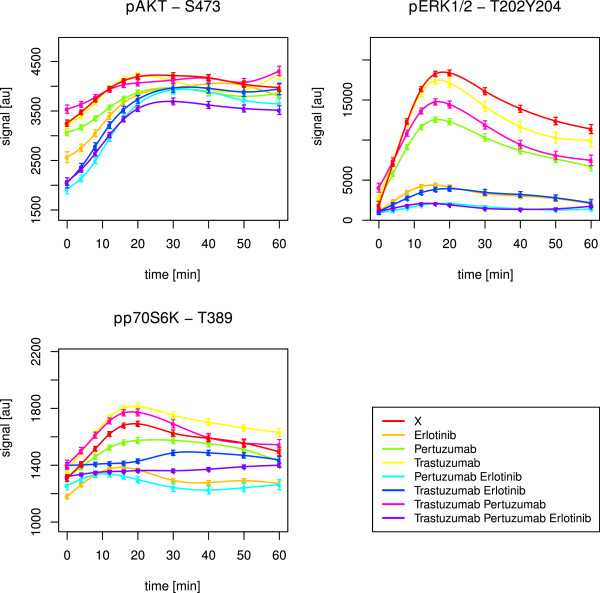
**HCC1954 short-term time courses of AKT, ERK1/2 and p70S6K.** The figure shows splines and related standard error bars of the measured RPPA data for AKT, ERK1/2 and p70S6K after combined EGF and HRG stimulation in the HCC1954 cell line. The measurements included ten time points up to 60 minutes. The different drug treatments are marked by different colours with ‘X’ denoting no drug treatment.

Stimulation with EGF and HRG should result in activation of ErbB-1 and ErbB-3, followed by dimerisation amongst ErbB members. This should initialise signalling cascades in the MAPK and PI3K pathways (Figure 2). Indeed, AKT, ERK1/2 and p70S6K got activated in all cell lines, which was revealed by simulations as well as observations in graphical analyses (Table 1, Figures 4, 5, 6).

As we were interested in identifying optimal drug treatments, Table 2 summarises the corresponding statistical results. Most of them were supported by the perturbation simulation results, corresponding to attractor states of AKT, ERK1/2 and p70S6K being zero. Four main conclusions were drawn from these results, which will be discussed in detail in the following subsections. Firstly, inhibition of PI3K signalling, reflected by downregulated AKT, required the combined treatment with erlotinib, pertuzumab and trastuzumab. Secondly, inhibition of the MAPK pathway, represented by ERK1/2, was reached with erlotinib alone in SKBR3 and HCC1954. BT474 additionally needed pertuzumab. Thirdly, the protein activity of p70S6K was influenced by both, PI3K and MAPK, pathways. The drug response differed between cell lines, indicating both pathways contribute to a different extent. Finally, the drug effect on PI3K signalling was much better in SKBR3 than in HCC1954, pointing to resistance in the latter cell line.

**Table 2:** Optimal drug treatment in short-term signalling

**Cell line**	**AKT**	**ERK1/2**	**p70S6K**
BT474	-	PE	PE
HCC1954	**PT**E	**E**	**P**E
SKBR3	**PTE**	**E**	**PTE**

The table summarises the optimal drug treatments for the short-term data, leading to inactive AKT, ERK1/2 and p70S6K, respectively. In case of a significant (p-value < 0.05) combined influence of both, drug treatment and time, on the protein signal intensity, a Wilcoxon rank sum test was conducted for the measurements at time point 60 minutes, testing for significantly (p-value < 0.05) smaller intensity values under the drug treatment compared to the control measurement. The drug treatment with the smallest p-value was considered as the optimal inhibitor. No inferred significant positive drug effect is denoted by ‘-’. The therapeutics erlotinib, trastuzumab and pertuzumab are abbreviated by their first letters. More than one letter denotes drug combinations. The growth factors EGF and HRG were added in combination to the cell lines and permanently active in the simulated perturbation conditions. The column *Cell line* holds the cell lines under consideration. The columns *AKT*, *ERK1/2* and *p70S6K* hold the optimal drug combinations for each target. If those were confirmed by the attractor states (0) of perturbation simulations, they are printed in bold.

#### Inhibition of PI3K signalling requires drug combinations

In SKBR3, the triple drug combination was most effective in inhibiting AKT (Figure 4, Table 2). In BT474, pertuzumab combined with erlotinib was most efficient, but AKT signalling was not fully suppressed as in SKBR3 (Figure 5). Statistically, we did not infer any significant positive drug effect in this cell line. Obviously, erlotinib in synergistic combination with at least pertuzumab was needed to block the ErbB-2 receptor and its heterodimerisation, mainly with ErbB-1, but also ErbB-3. The HRG activated ErbB-2/3 heterodimers and PI3K pathway in BT474, as revealed by the network reconstructions, might have prevented a potent drug efficacy.

Interestingly, BT474 and SKBR3 required pertuzumab. This drug was especially designed to prevent heterodimerisation with ErbB-2. The stimuli EGF and HRG together activate PI3K signalling by ErbB-2/3, ErbB-1/2 and ErbB-1/3 dimers (Figure 2). The need for pertuzumab combined with erlotinib indicated an important role of ErbB-1/2 dimers. This was supported by the fact, that in HCC1954 with dominant heterodimers of this type, as revealed by network reconstructions, none of the drugs was likewise efficient in inhibiting AKT (Figure 6). However, the optimal effect was revealed for the triple drug combination (Table 2). The simulations suggested pertuzumab alone or a combination of both monoclonal antibodies (Table 1). It has to be kept in mind, that the attractor states resembled a long-term steady state, which can differ from observations up to 60 minutes.

The perturbation simulations in BT474 did not lead to inactive AKT upon combined pertuzumab and erlotinib treatment. Instead, erlotinib alone or combined with trastuzumab was efficient (Table 1). Nevertheless, this supported the need for the small molecule inhibitor and a monoclonal antibody to suppress ErbB-2 induced PI3K signalling. In SKBR3, the attractor states confirmed the described optimal drug treatment to deactivate AKT. Trastuzumab, when applied alone, was the only treatment without a positive effect in the simulations (Table 1).

#### Inhibition of MAPK signalling requires erlotinib

Signalling through the MAPK pathway, represented by ERK1/2 activation, was efficiently inhibited by erlotinib alone in both, HCC1954 (Figure 6, Table 2) and SKBR3 (Figure 4, Table 2), cell lines. EGF activates the MAPK pathway via ErbB-1 homodimers and ErbB-1/2 heterodimers (Figure 2). Both are prevented by ErbB-1 inhibition via erlotinib, which was especially designed to target this receptor.

In BT474, pertuzumab plus erlotinib was required (Figure 5, Table 2). This was analogous to the situation in PI3K signalling.

HRG activates the MAPK pathway via ErbB-2/3 heterodimers (Figure 2). Obviously, BT474 needed the addition of the monoclonal antibody due to dominant ErbB-2/3 formation and activity. On the contrary, the other two cell lines just needed erlotinib alone. Here, in addition to the ErbB-1 dimers, the ligand-independent ErbB-2 homodimers might have driven ERK1/2 activation and could be inhibited by the small molecule inhibitor. Efficacy of erlotinib towards ErbB-2 dimers was previously mentioned by Schaefer et al. [61].

In BT474, the simulations resulted in active ERK1/2 states, resisting drug treatment (Table 1). In HCC1954 and SKBR3, the positive effect of erlotinib was supported by the simulations. The attractor states were additionally inactive for all other (combinatorial) drug treatments, but not trastuzumab alone.

#### p70S6K is influenced by both, PI3K and MAPK, pathways

The target p70S6K is upstream influenced by the PI3K as well as the MAPK pathway (Figure 2). Hence, p70S6K merges both pathways, leading to activation of RPS6 [60].

The three cell lines showed different pathway preferences. BT474 required the combination of pertuzumab and erlotinib to suppress p70S6K (Figure 5). On the contrary, in SKBR3 the triple drug combination was shown to be optimal (Table 2). Obviously, the effect was driven by erlotinib (Figure 4), which was supported by the attractor states of p70S6K (Table 1). This resembled the drug response of ERK1/2 and reflected a stronger influence by the MAPK pathway. In HCC1954, deactivation of p70S6K was reached via application of erlotinib combined with pertuzumab (Table 2). The treatment with erlotinib alone had a similar effect (Figure 6), while the simulations just confirmed a positive effect of pertuzumab (Table 1). Thus, this cell line seemed to be influenced by both, PI3K and MAPK, pathways.

These results were in line with the newly inferred edges in the network reconstructions. They pointed to a strong influence of PI3K in BT474 in contrast to a dominant MAPK pathway in SKBR3. HCC1954 was influenced by both pathways to a similar extent.

To follow up on the hypothesis that different pathways contribute to a different extent in individual cell lines, we tested correlation between the p70S6K time course and the ones of AKT and ERK1/2, respectively. In BT474, p70S6K correlated positively with AKT (p-value 0.01, Kendall’s *τ* estimate 0.64). In HCC1954, p70S6K correlated positively with both, AKT (p-value <2.22·10^−16^, Kendall’s *τ* estimate 0.69) and ERK1/2 (p-value <2.22·10^−16^, Kendall’s *τ* estimate 0.87). In SKBR3, p70S6K also correlated positively with both, AKT (p-value 0.05, Kendall’s *τ* estimate 0.51) and ERK1/2 (p-value 0.02, Kendall’s *τ* estimate 0.6), with a stronger tendency towards MAPK signalling. The correlation was not as convincing as in the other two cell lines. One could speculate, that the dominance of the MAPK pathway in SKBR3 cells was not as strong as the dominance of the PI3K pathway in BT474. This was supported by the reconstructed networks. They revealed downstream effects of MAPK signalling in SKBR3, while they revealed hyperactive ErbB-2/3 dimers in BT474. The dimers drive PI3K already at the receptor layer, and especially ErbB-2/3 dimers are regarded as the most potent heterodimer [29].

#### Drug resistance in HCC1954 regarding the PI3K pathway

In HCC1954, the inferred optimal treatment against AKT signalling with the triple drug combination was not convincing (Figure 6). Analogously, Henjes et al. did not monitor any positive drug effect on AKT under EGF application alone [28]. However, the simulations suggested pertuzumab alone or a combination of both monoclonal antibodies to inhibit AKT phosphorylation. In principle, divergence of simulations from experimental observations can be expected, as the simulated steady state of the system does not necessarily have to be reached after the measured period of time. Anyhow, the apparent resistance here pointed to a hyperactive PI3K pathway which was explainable by the newly inferred HCC1954 edges described in the previous subsection. They represented feedback loops, hyperactive ErbB-1/2 heterodimers and pathway crosstalk. On the contrary, in SKBR3, the triple drug combination worked well, as described before. The simulations even predicted efficacy of every other drug (combination) apart from trastuzumab alone. The drug efficacy towards AKT in this cell line could be explained by the fact that the two reconstructed interactions in SKBR3 mainly promoted the MAPK instead of the PI3K pathway.

The regulation of AKT activity under drug influence, highly diverging in HCC1954 and SKBR3, attracted our attention. Therefore we intended testing for edgetic mutations, as discussed by Zhong et al. [62], leading to AKT gain-of-function in HCC1954. Such mutations, perturbing not a node but an edge of a network, are speculated to have deeper impact on phenotypic manifestation of a disease. In detail, we removed each of the AKT stimulating edges outgoing from p70S6K, PDK1, mTOR and ErbB-3, alone or in all possible eleven combinations. We then computed the attractor states for the modified networks in HCC1954.

Removal of the connections of mTOR, PDK1 and ErbB-3 alone or combined had no influence on improving drug effects, i.e. AKT just got inactive under pertuzumab treatment. Involvement of p70S6K →AKT in the withdrawal process led to much better results. Removed alone or in double combinations with the aforementioned edges, as well as in the two triple combinations containing mTOR, AKT was deactivated under all drug treatments, but not yet trastuzumab alone. Finally, simultaneous removal of the outgoing connections from p70S6K, ErbB-3 and PDK1 with or without mTOR, turned out as the only combination enabling potency of all possible drug combinations, including trastuzumab alone. This hinted at a less strong impact of mTOR on AKT here, but indicated synergistic drug resistance potential of p70S6K, ErbB-3 and PDK1, also due to the newly inferred edges.

### Long-term signalling network reconstruction

The reconstructed long-term signalling networks per cell line are displayed in the Additional file 6. Additional file 5 lists the equivalent Boolean logical interaction rules. Compared to the prior network, most of the newly inferred edges were individual for each cell line, but HCC1954 shared ErbB-1 →ERK1/2 with SKBR3, for example. This seemed to be an indirect edge via cRAF, as represented in the prior network. Besides activating connections, also inhibiting ones and edge deletions occurred. For HCC1954, ten new interactions were reconstructed, while two were deleted. In BT474, nine new links were added, and one edge was deleted. In SKBR3, we inferred 20 new connections and one deletion, namely the removal of p53 activation via p38, bearing oncogenic risk [63,64].

In contrast to the short-term networks, new feedback loops were reconstructed in every cell line, not exclusively in HCC1954. In HCC1954, the mutual activation between p53 and RB established such a feedback mechanism. For SKBR3 we even inferred two edges, each forming feedback loops. Contrary to HCC1954, p53 inhibited RB. The second loop connection was inhibition of ErbB-3 by AKT, pointing to a negative feedback against PI3K signalling [65-67].

In HCC1954, the newly inferred edges Cyclin B1 →AKT and ErbB-3 →ErbB-1 contributed to PI3K signalling, of which the latter was explainable as heterodimers. The newly inferred edge cJUN →ErbB-1 in HCC1954 also indicated raised activity of ErbB-1. Interestingly, in SKBR3 we conducted an inhibiting edge from Cyclin B1 to AKT but instead an activating one to ERK1/2, contributing to MAPK signalling, which was also stated by Abrieu et al. [68]. Another new edge in HCC1954 involved a cell cycle player, i.e. activation of Cyclin D1 by p70S6K [69]. Accordingly, we inferred RPS6 →Cyclin D1 in BT474, with RPS6 as downstream target of p70S6K. In SKBR3, the edge p70S6K →Cyclin B1 was reconstructed. A further interesting new activating edge in HCC1954 led from RB to TSC2, while we inferred a reversed inhibition in SKBR3. Searle et al. discussed targeting RB deficient cancers by deactivating TSC2 [70].

Two novel interactions in BT474 activated Cyclin B1, arising from ErbB-1 and ErbB-3, respectively, which meant that mitosis was driven by ErbB-1/3 dimers in this cell line. This indicated a hyperactive PI3K pathway, as revealed in the short-term case.

In SKBR3, we reconstructed an outgoing edge from the artificial network stimulus S, representing full growth medium, activating AKT. This could be explained as strong activation of AKT, driving PI3K signalling in this cell line. The new edges ErbB-2 →TSC2 and ErbB-3 →PRAS had to be interpreted as indirect effects, too. They pointed to activity of ErbB-2/3 dimers, feeding into both, MAPK and PI3K, pathways. The edge ErbB-2 →TSC2 could imply an oncogenic role of TSC2. Liu et al. discussed a context dependent functionality of TSC2 [71].

### Perturbation simulations on long-term networks

Similarly to the perturbation simulations for the short-term networks, we performed those for the long-term networks under all eight initial state combinations of the therapeutics erlotinib, pertuzumab and trastuzumab. Also here, all inferred attractors were simple and consisted of one steady-state. Table 3 contains the simulation results for the attractors of the RPS6 and RB proteins, as those are key players in cell growth and proliferation and mainly comparable to the experimental results of Henjes et al. [28] for HCC1954 and SKBR3. We analysed the attractor states of AKT and ERK1/2, too, but the results are not explicitly listed, since they mostly resembled the ones of RPS6.

**Table 3:** Attractor states of long-term perturbation simulations

	**BT474**				**HCC1954**				**SKBR3**			
**Simulation**	**RPS6**		**RB**		**RPS6**		**RB**		**RPS6**		**RB**	
	**A**	**E**	**A**	**E**	**A**	**E**	**A**	**E**	**A**	**E**	**A**	**E**
X	**1**		0	1	**1**		0	1	**1**		0	1
E	0	1	**0**		0	1	**0**		0	1	**0**	
P	0	-	0	-	0	-	0	-	0	-	0	-
T	1	-	0	-	1	-	0	-	1	-	0	-
E, P	0	1	**0**		0	1	**0**		0	1	**0**	
E, T	0	1	**0**		0	1	**0**		**0**		**0**	
P, T	**0**		**0**		0	1	0	1	**0**		**0**	
E, P, T	0	1	**0**		0	1	0	1	**0**		**0**	

The therapeutics erlotinib, trastuzumab and pertuzumab, abbreviated by first letters, that were permanently active in the simulated perturbation conditions besides the stimulus S, standing for the full growth medium, are stored in the column *Simulation*. No simulated drug treatment is denoted by ‘X’. The *A* columns hold the attractor states of the proteins RPS6 and RB associated with the perturbations. The *E* columns contain the protein activity status, statistically deduced from the experimental data. In case of a significant (p-value < 0.05) combined influence of both, drug treatment and time, on the protein signal intensity, a Wilcoxon rank sum test was conducted for the measurements at time point 30 hours. The drug treatments leading to significantly (p-value < 0.05) smaller intensity values compared to the control measurement ‘X’ were considered as efficient inhibitors, resulting in a table entry of zero. Lacking comparable experiments is labelled as ‘-’, while consistency between simulations and experimental observations is printed in bold.

The control measurements without any drug treatment should result in activation of ErbB members and dimerisation events, promoting cell growth and proliferation. In fact, this was expressed as reasonable activation of AKT, ERK1/2 and RPS6 in all cell lines, which held for simulations as well as experimental observations. In contrast, the attractor states of RB were inactive in all cell lines (Table 3). Actually, a continuously rising stimulation effect over 30 hours was not observed for HCC1954 and SKBR3 by Henjes et al. [28] either.

The attractor states of RPS6 and RB were identical in all cell lines (Table 3). All drugs, except trastuzumab under stimulation alone, led to inactive attractor states of RPS6. This was also the case for ERK1/2 in all cell lines, as well as AKT in BT474 and HCC1954. In SKBR3, the attractor states of AKT were just inactive without the stimulus. All therapeutics, including trastuzumab, resulted in deactivated attractor states of RB. The statistically inferred drug effects for AKT, ERK1/2, RB and RPS6 were slightly different. Table 4 summarises the optimal drug combinations, confirming and extending the observations of Henjes et al. [28]. Most of them were supported by the perturbation simulation results, corresponding to attractor states of AKT, ERK1/2, RB and RPS6 being zero.

**Table 4:** Optimal drug treatment in long-term signalling

**Cell line**	**AKT**	**ERK1/2**	**RB**	**RPS6**
BT474	-	**TE**	**E**	**TP**
HCC1954	**E**	**TE**	**E**	-
SKBR3	PTE	**TE**	**TE**	**TE**

The table summarises the optimal drug treatments for the long-term data, leading to inactive AKT, ERK1/2, RB and RPS6, respectively. In case of a significant (p-value < 0.05) combined influence of both, drug treatment and time, on the protein signal intensity, a Wilcoxon rank sum test was conducted for the measurements at time point 30 hours, testing for significantly (p-value < 0.05) smaller intensity values under the drug treatment compared to the control measurement. The drug treatment with the smallest p-value was considered as the optimal inhibitor. No inferred significant positive drug effect is denoted by ‘-’. The therapeutics erlotinib, trastuzumab and pertuzumab are abbreviated by their first letters. More than one letter denotes drug combinations. The column *Cell line* holds the cell lines under consideration. The columns *AKT*, *ERK1/2*, *RB* and *RPS6* hold the optimal drug combinations for each target. If those were confirmed by the attractor states (0) of perturbation simulations, they are printed in bold.

#### The optimal long-term drug response for AKT and ERK1/2 confirms short-term observations

**Figure 7:**
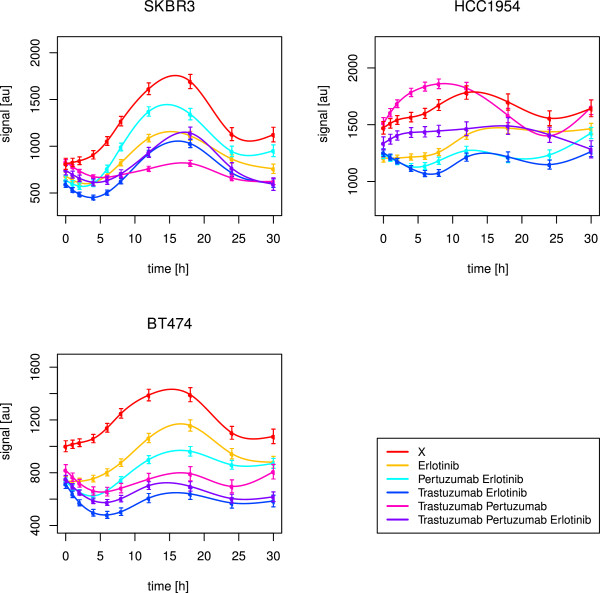
**Long-term time courses of AKT for all cell lines.** The figure shows splines and related standard error bars of the measured RPPA data for AKT in all cell lines. The measurements included ten time points up to 30 hours. The different drug treatments are marked by different colours with ‘X’ denoting no drug treatment.

As shown in Figure 7, the best drug response in BT474 and HCC1954 regarding AKT was yielded for a combination of trastuzumab and erlotinib. Statistically, we inferred no positive effect in BT474 at all, which is explainable by the fact that we just considered a combined effect of drug treatment and time. Although the time courses of AKT signalling with and without the drug treatment were differing in the intensity strength, the signalling profiles were similar. This parallel shift indicated no time effect. Instead, the group effect was significant (p-value <2·10^−16^).

This was also the explanation, why we detected erlotinib, but not the combination with trastuzumab, as the optimal treatment in HCC1954 (Table 4). In SKBR3, we inferred the triple drug combination as the optimal one, but the combination of both monoclonal antibodies alone also had a significant effect over time (Figure 7). Hence, like in the short-term results, a drug combination was required to suppress PI3K signalling, here with an obvious need for trastuzumab. For BT474 and HCC1954, this was supported by the simulation results, in which trastuzumab alone had no effect, but was efficient within drug combinations. In HCC1954, even the best drug response was not convincing (Figure 7), pointing to a dominant PI3K pathway, as revealed in the short-term analysis. Interestingly, SKBR3 showed a strong activation peak of AKT phosphorylation between 8 and 18 hours (Figure 7), which was just suppressed under combined application of trastuzumab and pertuzumab. We revealed a positive correlation with ERK1/2 (p-value 0.02, Kendall’s *τ* estimate 0.6) and RPS6 (p-value 0.01, Kendall’s *τ* estimate 0.64). The reconstructed edges S →AKT and ErbB-1 →ERK1/2 in SKBR3 indicated strong activation of AKT and ERK1/2. In addition to the prior network, in which AKT and ERK1/2 fed into RPS6 phosphorylation via p70S6K, some of the novel edges pointed to a positive feedback from p70S6K or RPS6 to ERK1/2. The feedback from p70S6K via Cyclin B1, for example, was expressed by the edges p70S6K →Cyclin B1 and Cyclin B1 →ERK1/2. Compared to the short-term results, indicating a dominant MAPK pathway, this long-term observation indicated strong signalling via both, PI3K and MAPK, pathways in SKBR3.

**Figure 8:**
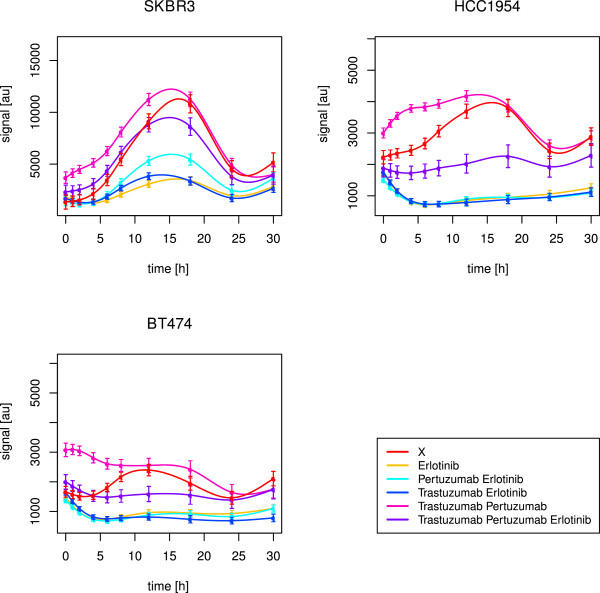
**Long-term time courses of ERK1/2 for all cell lines.** The figure shows splines and related standard error bars of the measured RPPA data for ERK1/2 in all cell lines. The measurements included ten time points up to 30 hours. The different drug treatments are marked by different colours with ‘X’ denoting no drug treatment.

As displayed in Figure 8, erlotinib alone or in combination with trastuzumab showed the optimal effect against ERK1/2 in all of the three cell lines. This was in line with the short-term observations, and confirmed by the perturbation simulations. Statistically, the most potent drug effect was yielded with the combination of erlotinib and trastuzumab (Table 4).

#### Quick drug response for RPS6 and delayed response for RB

**Figure 9:**
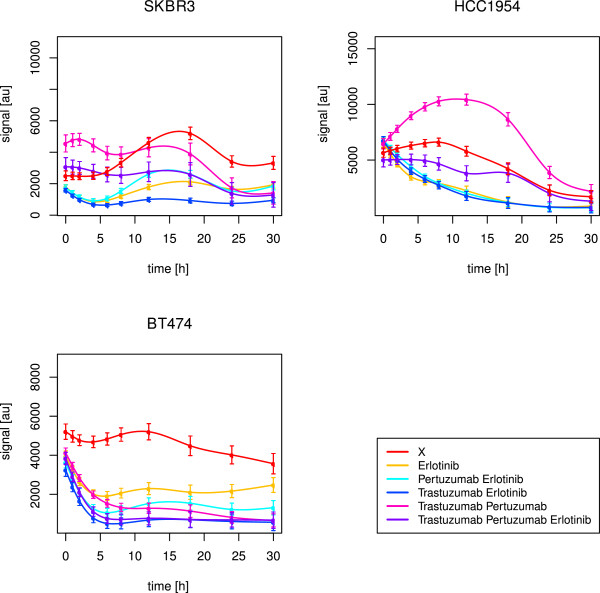
**Long-term time courses of RPS6 for all cell lines.** The figure shows splines and related standard error bars of the measured RPPA data for RPS6 in all cell lines. The measurements included ten time points up to 30 hours. The different drug treatments are marked by different colours with ‘X’ denoting no drug treatment.

**Figure 10:**
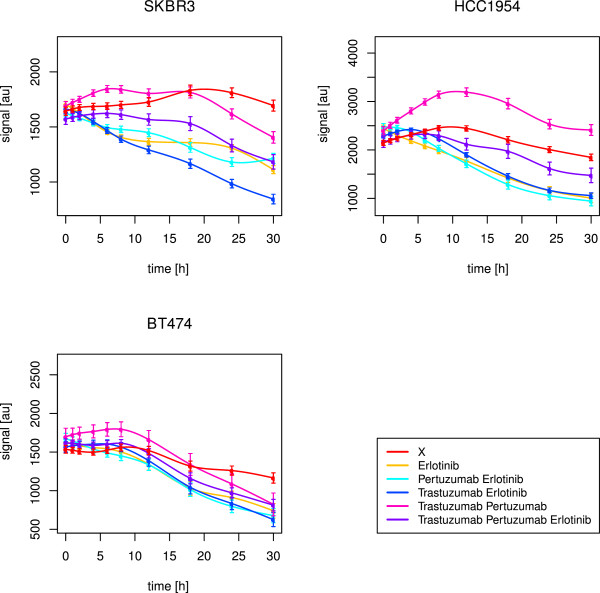
**Long-term time courses of RB for all cell lines.** The figure shows splines and related standard error bars of the measured RPPA data for RB in all cell lines. The measurements included ten time points up to 30 hours. The different drug treatments are marked by different colours with ‘X’ denoting no drug treatment.

As shown in Figure 9, in BT474, the simulation based predicted efficacy of erlotinib alone to counteract RPS6 (Table 3) was not as convincing as in case of drug combinations. A combination of pertuzumab and trastuzumab worked best (Table 4). For RB, the simulated drug effects in BT474 resembled the observed ones (Table 3, Figure 10), with a positive effect of all measured drug treatments. Erlotinib was inferred as the optimal treatment (Table 4). Though, the drug impact unfolded not before 18 hours.

In HCC1954, it was the combination of both monoclonal antibodies, that failed in deactivating RPS6 (Figure 9), while the simulations predicted trastuzumab alone to fail (Table 3). The graphical observations were similar for RB (Figure 10). The newly inferred edges ErbB-3 →ErbB-1 and cJUN →ErbB-1 in HCC1954 explained the necessity for erlotinib against ErbB-1 dimers. The positive impact of erlotinib, the optimal treatment against RB signalling (Table 4), was supported by simulations. However, it did not unfold before 12-18 hours, in case of RB as well as RPS6. Regarding RPS6, no significant effect was detected for HCC1954 (Table 4).

According to Henjes et al. [28], in SKBR3 erlotinib and all therapeutic combinations helped to suppress RPS6, which was supported by the simulations (Table 3). As shown in Figure 9, the combination of trastuzumab and erlotinib was the only one, that revealed its continuous inhibiting effect already after one hour. This combined treatment was also statistically inferred as the optimal one (Table 4). The same combination was optimal with respect to RB activity, which was also in line with the simulations. Here, analogously to BT474 and HCC1954, the drug effect did not appear before 18 hours (Figure 10).

As the combination of trastuzumab and erlotinib was efficient in all of the three cell lines against RPS6 as well as RB phosphorylation, we further analysed target correlations under this drug combination to explain the different rapidness of drug responses.

In BT474, RB positively correlated with Cyclin B1 (p-value 0.02, Kendall’s *τ* estimate 0.6), while RPS6 positively correlated with ERK1/2 (p-value 0.02, Kendall’s *τ* estimate 0.6). Obviously, RPS6 was mainly stimulated by the MAPK pathway, which was efficiently inhibited by the combination of trastuzumab and erlotinib in a fast manner. On the contrary, RB seemed to be influenced by Cyclin B1. The newly reconstructed edges ErbB-1 →Cyclin B1 and ErbB-3 →Cyclin B1 supported hyperactivity of Cyclin B1, driven by ErbB-1/3 heterodimers. In SKBR3, RB negatively correlated with PRAS (p-value 0.05, Kendall’s *τ* estimate -0.51) and TSC2 (p-value 0.03, Kendall’s *τ* estimate -0.56), while RPS6 positively correlated with AKT (p-value 0.03, Kendall’s *τ* estimate 0.56) and ERK1/2 (p-value 0.02, Kendall’s *τ* estimate 0.6). Obviously, like in BT474, RPS6 was mainly activated through the MAPK pathway. Interestingly, RB seemed to require inhibition via PRAS or TSC2. The latter was confirmed via one of the novel edges in SKBR3, namely inhibition of RB by TSC2. In addition, PRAS as well as TSC2 seemed to be especially active in this cell line with regard to the new edges ErbB-3 →PRAS and ErbB-2 →TSC2.

In HCC1954, the drug response was not only delayed for RB, but also for RPS6, which was in line with the positive correlation with RB (p-value <2.22·10^−16^, Kendall’s *τ* estimate 0.73). Like in BT474, Cyclin B1 seemed to be a driving force, since both, RPS6 (p-value 0.03, Kendall’s *τ* estimate 0.56) and RB (p-value <2.22·10^−16^, Kendall’s *τ* estimate 0.82) positively correlated with this target. The new edge Cyclin B1 →AKT supported special activation of RPS6 via PI3K signalling, leading to a delayed drug response. Interestingly, we revealed negative correlations, as observed for SKBR3. In HCC1954, RPS6 and RB correlated with BAX (p-value 0.03, Kendall’s *τ* estimate -0.56) and FoxO1/3a (p-value 0.05, Kendall’s *τ* estimate -0.51), pointing to a delayed inhibition of RPS6 and RB via BAX or FoxO1/3a.

## Conclusions

Using a combination of reverse and forward engineering techniques, we focused on deregulated protein interactions in the ErbB network in a Boolean modelling framework. The reconstructed hypothetical networks revealed individual protein interactions contributing to signalling pathway preferences as well as drug resistance via feedback loops, pathway crosstalk or hyperactive heterodimers. While this reverse engineering focused on the network edges, we concentrated in the subsequent forward engineering step on the network nodes. The perturbation simulations for AKT, ERK1/2, RB and RPS6 mainly confirmed our graphical and statistical analyses as well as the observations of Henjes et al. [28] regarding (combinatorial) drug efficacy. However they have to be interpreted as an independent, more prospective investigation, because stable system states do not necessarily have to be reached in temporally limited observations.

In the first step, the combined Boolean modelling approach revealed the mechanisms underlying individual drug response. In the second step, it predicted the network propagation effects on protein activity, and hence the drug response itself.

One major finding is, that different breast cancer phenotypes seem to be driven by specific pathway preferences in the ErbB network. This leads to individual drug response, requiring different therapeutic treatments. The perturbation simulations revealed a more diverse drug response in short-term than in long-term signalling, which stresses the importance of early intervention at the top level layer of the signalling network.

Another interesting aspect is to combine edge and node perturbations in Boolean network models to reveal edgetic mutations, as we did in the HCC1954 cell line for AKT.

Basic molecular research, embedded in a Boolean modelling framework here, composes a first step to gain insight into individual mechanisms of drug response or resistance mechanisms in breast cancer. Especially, the proteomic signalling interplay directly effects tumour development and represents a promising target in cancer therapy, which has to be understood in more detail in the future.

## Abbreviations

DDEPN: Dynamic deterministic effects propagation networks; EGF: Epidermal growth factor; EGFR: Epidermal growth factor receptor; GEO: Gene expression omnibus; HER2: Human epidermal growth factor receptor 2; HMM: Hidden Markov model; HRG: Heregulin; MAPK: Mitogen-activated protein kinase; MCMC: Markov chain Monte Carlo; NIR: Near infrared; PI3K: Phosphoinositide 3-kinase; RPPA: Reverse phase protein array; RTK: Receptor tyrosine kinase.

## Competing interests

The authors declare that they have no competing interests.

## Authors’ contributions

FH performed the RPPA measurements under supervision of UK and was mainly involved in target selection for the modelling approach. JS was involved in discussing the conducted RPPA experiments. CB and TB developed the applied network reconstruction algorithm and participated in planning the modelling procedure. TB and SvdH initiated the simulation study concepts. SvdH carried out the literature research, network reconstructions, perturbation simulations followed by associated analyses, and finally drafting the manuscript. All authors edited, read and approved the final manuscript.

## Supplementary Material

Additional file 1 **Proteins and phosphorylation sites involved in RPPA measurements.** The tables show the proteins and phosphorylation sites involved in RPPA short- and long-term measurements. The antibody catalogue numbers and providing companies are mentioned in brackets. For BT474, no experimental short-term data under EGF or HRG stimulation were available for PDK1. In case of total protein measurements, the column *Phosphosite* remains empty (‘-’) apart from the antibody number and supplier name. Click here for file

Additional file 2 **Literature references for the prior networks of short- and long-term signalling.** The interactions between proteins are listed line by line in the tables. The column *Protein* denotes the source of the connection with the sink called *Target*. The interaction (*Type*) is encoded numerically, i.e. activation is marked by 1, while inhibition is labelled with 2, e.g. AKT activates mTOR. The column *Reference* specifies the supportive publication. Click here for file

Additional file 3 **Workflow of MCMC-based network structure inference.** The *inhibMCMC* procedure of the *ddepn* package was run with *maxIter* = 50,000 in 10 parallel runs. The results of the 25,000 iterations after the burn-in phase were merged into one consensus network. It was applied for short- and long-term data separately per cell line, leading to six consensus networks. The figure is based on [6,47]. Click here for file

Additional file 4 **Edge confidences for the reconstructed networks of the three cell lines per time course.** In each of the ten MCMC runs, activation and inhibition edges were sampled. The percentage, i.e. the confidence, of sampled activation (red) and inhibition (blue) edges in the 25,000 iterations after the burn-in phase are depicted in the boxes. The sink nodes are displayed in each panel, while the activating, inhibiting or missing influence of the source nodes is shown column-wise in the red, blue or missing boxes. The source node names are displayed at the x-axis with additional indicators, where ‘-’ refers to an inhibiting influence and ‘+’ is related to activation. The x-axis of the short-term plots is labelled as ‘AKT, E, EGF, ERBB1, ERBB2, ERBB3, ERK1/2, HRG, MEK1/2, mTOR, P, p70S6K, PDK1, PKC *α*, PLC *γ*, T’. The x-axis of the long-term plots is labelled as ‘AKT, BAX, cJUN, cRAF, CyclinB1, CyclinD1, E, ERBB1, ERBB2, ERBB3, ERK1/2, FOXO1/3a, GSK3 *α*/ *β*, NF- *κ*B, P, p38, p53, p70S6K, PRAS, PTEN, RB, S, RPS6, T, TSC2’. An activating edge in the consensus network, as described in Additional file 3, means that the sampled activating edges have a significantly higher confidence value than the inhibiting ones. As self-loops and ingoing edges to the drug or growth factor nodes were not allowed during inference, the respective confidences are zero. Click here for file

Additional file 5 **Boolean interaction rules for the components of the short- and long-term signalling networks.** The tables contain the rules that arose from network reconstructions based on short- and long-term RPPA data of BT474, HCC1954 and SKBR3. The three drug names erlotinib, trastuzumab and pertuzumab are abbreviated via their first letters. For the long-term networks, the stimulus is denoted by *S*. Symbols are interpretable in the following way: & ≡ AND, ∨≡ OR and ! ≡ NOT. Click here for file

Additional file 6 **Reconstructed long-term signalling networks.** The figure displays the reconstructed long-term signalling networks for BT474, HCC1954 and SKBR3. Target proteins are represented as rectangles with stimulus and drugs coloured in red. The three drug names erlotinib, trastuzumab and pertuzumab are abbreviated via their first letters. Stimulation via full growth medium is denoted by *S*. Solid arrows denote activating interactions while dashed ones represent inhibitions. Click here for file
