# Supplementary material for: Boolean ErbB network reconstructions and perturbation simulations reveal individual drug response in different breast cancer cell lines
Source: BMC Syst Biol. 2014 Jun 25;8:75. doi: 10.1186/1752-0509-8-75 (PMC4087127; doi:10.1186/1752-0509-8-75)
Supplement: Additional file 1 — Proteins and phosphorylation sites involved in RPPA measurements. The tables show the proteins and phosphorylation sites involved in RPPA short- and long-term measurements. The antibody catalogue numbers and providing companies are mentioned in brackets. For BT474, no experimental short-term data under EGF or HRG stimulation were available for PDK1. In case of total protein measurements, the column Phosphosite remains empty (‘-’) apart from the antibody number and supplier name. [file 1752-0509-8-75-S1.pdf]

## Additional file 1 — Proteins and phosphorylation sites involved in RPPA measurements

The tables show the proteins and phosphorylation sites involved in RPPA short- and long-term measurements. The antibody catalogue numbers and providing companies are mentioned in brackets. For BT474, no experimental short-term data under EGF or HRG stimulation were available for PDK1. In case of total protein measurements, the column *Phosphosite* remains empty ('-') apart from the antibody number and supplier name.

| **Short-term data** | | |
|---|---|---|
| Protein | Phosphosite | |
| | BT474 | HCC1954 & SKBR3 |
| AKT | S473 (9271, CST) | |
| ERBB1 | Y1086 (2220, CST) | Y1068 (2236, CST) |
| ERBB2 | Y1221/Y1222 (2243, CST) | Y1248 (M06229, Millipore) |
| ERBB3 | Y1289 (4791, CST) | |
| ERK1/2 | T202/Y204 (9106, CST) | T202/Y204 (4370, CST) |
| MEK1/2 | S217/S221 (M7683, Sigma) | |
| mTOR | S2481 (M09343, Millipore) | S2448 (2971, CST) |
| p70S6K | T389 (9206, CST) | |
| PDK1 | *no measurement* | S241 (3438, CST) |
| PKC$\alpha$ | S657/Y658 (ab23513, abcam) | |
| PLC$\gamma$ | S1248 (4510, CST) | |

| Long-term data | |
|---|---|
| Protein | Phosphosite |
| | BT474, HCC1954 & SKBR3 |
| AKT | S473 (9271, CST) |
| BAX | - (2772, CST) |
| cJUN | S63 (BD558036, BD) |
| cRAF | S338 (9427, CST) |
| Cyclin B1 | - (DLN09016, Dianova) |
| Cyclin D1 | - (2922, CST) |
| ERBB1 | Y1173 (4407, CST) |
| ERBB2 | Y1248 (M06229, Millipore) |
| ERBB3 | Y1289 (4791, CST) |
| ERK1/2 | T202/Y204 (4370, CST) |
| FoxO1/3a | T24/T32 (9464, CST) |
| GSK3$\alpha/\beta$ | Y279/Y216 (E23091, Epitomics) |
| NF-$\kappa$B | S536 (3033, CST) |
| p38 | T180Y182 (9211, CST) |
| p53 | - (sc126, Santa-Cruz) |
| p70S6K | T389 (9206, CST) |
| PRAS | T246 (2997, CST) |
| PTEN | - (9552, CST) |
| RB | S807/S811 (9308, CST) |
| RPS6 | S235/S236 (4858, CST) |
| TSC2 | T1462 (3617, CST) |
