# Supplementary material for: Boolean ErbB network reconstructions and perturbation simulations reveal individual drug response in different breast cancer cell lines
Source: BMC Syst Biol. 2014 Jun 25;8:75. doi: 10.1186/1752-0509-8-75 (PMC4087127; doi:10.1186/1752-0509-8-75)
Supplement: Additional file 2 — Literature references for the prior networks of short- and long-term signalling. The interactions between proteins are listed line by line in the tables. The column Protein denotes the source of the connection with the sink called Target. The interaction (Type) is encoded numerically, i.e. activation is marked by 1, while inhibition is labelled with 2, e.g. AKT activates mTOR. The column Reference specifies the supportive publication. [file 1752-0509-8-75-S2.pdf]

Additional file 2 — Literature references for the prior networks of short- and long-term signalling

The interactions between proteins are listed line by line in the tables. The column *Protein* denotes the source of the connection with the sink called *Target*. The interaction (*Type*) is encoded numerically, i.e. activation is marked by 1, while inhibition is labelled with 2, e.g. AKT activates mTOR. The column *Reference* specifies the supportive publication.

**Short-term prior network**			
Protein	Target	Type	Reference
AKT	mTOR	1	[1]
AKT	p70S6K	1	[1]
EGF	ERBB1	1	[2–4]
ERBB1	MEK1/2	1	[5–7]
ERBB1	PDK1	1	[7–10]
ERBB1	PLCγ	1	[11, 12]
ERBB2	MEK1/2	1	[6, 7]
ERBB2	PDK1	1	[7, 8, 13]
ERBB2	PLCγ	1	[14]
ERBB3	AKT	1	[10, 15–17]
ERBB3	MEK1/2	1	[4, 6]
ERBB3	PDK1	1	[8, 10, 15]
ERBB3	PLCγ	1	[10, 11, 15–17]
ERK1/2	p70S6K	1	[18]
Erlotinib	ERBB1	2	[19]
Erlotinib	ERBB2	2	[3, 19]
Erlotinib	ERBB3	2	[4, 19]
HRG	ERBB3	1	[20]
MEK1/2	ERK1/2	1	[21]
mTOR	AKT	1	[1]
mTOR	p70S6K	1	[22]
PDK1	AKT	1	[23]
Pertuzumab	ERBB1	2	[3, 24]
Pertuzumab	ERBB2	2	[24]
Pertuzumab	ERBB3	2	[4, 24]
PLCγ	ERBB1	1	[12]
PLCγ	PKCα	1	[25]
Trastuzumab	ERBB2	2	[24]

Long-term prior network			
Protein	Target	Type	Reference
AKT	Cyclin D1	1	[26, 27]
AKT	FoxO1/3a	2	[28, 29]
AKT	p38	1	[30]
AKT	PRAS	2	[31]
AKT	TSC2	2	[27, 32]
cJUN	Cyclin D1	1	[33]
cRAF	ERK1/2	1	[6, 21]
Cyclin B1	p53	1	[34]
Cyclin D1	RB	2	[35]
ERBB1	AKT	1	[36, 37]
ERBB1	cRAF	1	[5, 7]
ERBB2	AKT	1	[7, 13, 16, 17]
ERBB2	cRAF	1	[5, 7, 38]
ERBB3	AKT	1	[10, 15–17]
ERK1/2	cRAF	2	[39]
ERK1/2	Cyclin D1	1	[40]
ERK1/2	p70S6K	1	[18]
ERK1/2	TSC2	2	[41]
Erlotinib	ERBB1	2	[19]
Erlotinib	ERBB2	2	[3, 19]
Erlotinib	ERBB3	2	[4, 19]
GSK3α/β	cJUN	2	[42]
GSK3α/β	Cyclin D1	2	[27]
GSK3α/β	PTEN	2	[43]
GSK3α/β	TSC2	2	[27]
p38	p53	1	[44]
p53	Cyclin B1	2	[45]
p53	GSK3α/β	1	[46]
p70S6K	RPS6	1	[22]
Pertuzumab	ERBB1	2	[3, 24]
Pertuzumab	ERBB2	2	[24]
Pertuzumab	ERBB3	2	[4, 24]
PRAS	p70S6K	2	[22, 31]
PTEN	AKT	2	[47]
PTEN	p53	1	[48]
RB	p53	1	[49]
Trastuzumab	ERBB2	2	[24]
TSC2	p70S6K	2	[50]

References

1. Tato I, Bartrons R, Ventura F, Rosa JL: **Amino acids activate mammalian target of rapamycin complex 2 (mTORC2) via PI3K/Akt signaling**. *The Journal of biological chemistry* 2011, **286**(8):6128–6142. [PMID: 21131356].
2. Wong L, Deb TB, Thompson SA, Wells A, Johnson GR: **A differential requirement for the COOH-terminal region of the epidermal growth factor (EGF) receptor in amphiregulin and EGF mitogenic signaling**. *The Journal of biological chemistry* 1999, **274**(13):8900–8909. [PMID: 10085134].
3. Johannessen LE, Haugen KE, østvold AC, Stang E, Madshus IH: **Heterodimerization of the epidermal-growth-factor (EGF) receptor and ErbB2 and the affinity of EGF binding are regulated by different mechanisms**. *The Biochemical journal* 2001, **356**(Pt 1):87–96. [PMID: 11336639].
4. Alroy I, Yarden Y: **The ErbB signaling network in embryogenesis and oncogenesis: signal diversification through combinatorial ligand-receptor interactions**. *FEBS letters* 1997, **410**:83–86. [PMID: 9247128].
5. Hallberg B, Rayter SI, Downward J: **Interaction of Ras and Raf in intact mammalian cells upon extracellular stimulation**. *The Journal of biological chemistry* 1994, **269**(6):3913–3916. [PMID: 8307946].
6. Wu X, Noh SJ, Zhou G, Dixon JE, Guan KL: **Selective activation of MEK1 but not MEK2 by A-Raf from epidermal growth factor-stimulated Hela cells**. *The Journal of biological chemistry* 1996, **271**(6):3265–3271. [PMID: 8621729].
7. Zhan L, Xiang B, Muthuswamy SK: **Controlled activation of ErbB1/ErbB2 heterodimers promote invasion of three-dimensional organized epithelia in an ErbB1-dependent manner: implications for progression of ErbB2-overexpressing tumors**. *Cancer research* 2006, **66**(10):5201–5208. [PMID: 16707444].
8. Xin M, Gao F, May WS, Flagg T, Deng X: **Protein kinase Czeta abrogates the proapoptotic function of Bax through phosphorylation**. *The Journal of biological chemistry* 2007, **282**(29):21268–21277. [PMID: 17525161].
9. Zheng X, Jiang F, Katakowski M, Zhang ZG, Lu Qe, Chopp M: **ADAM17 promotes breast cancer cell malignant phenotype through EGFR-PI3K-AKT activation**. *Cancer biology & therapy* 2009, **8**(11):1045–1054. [PMID: 19395875].
10. Scaltriti M, Baselga J: **The epidermal growth factor receptor pathway: a model for targeted therapy**. *Clinical cancer research: an official journal of the American Association for Cancer Research* 2006, **12**(18):5268–5272. [PMID: 17000658].
11. Wang Y, Wu J, Wang Z: **Akt binds to and phosphorylates phospholipase C-gamma1 in response to epidermal growth factor**. *Molecular biology of the cell* 2006, **17**(5):2267–2277. [PMID: 16525023].
12. Tvorogov D, Carpenter G: **EGF-dependent association of phospholipase C-gamma1 with c-Cbl**. *Experimental cell research* 2002, **277**:86–94. [PMID: 12061819].
13. Gout I, Dhand R, Panayotou G, Fry MJ, Hiles I, Otsu M, Waterfield MD: **Expression and characterization of the p85 subunit of the phosphatidylinositol 3-kinase complex and a related p85 beta protein by using the baculovirus expression system**. *The Biochemical journal* 1992, **288 (Pt 2)**:395–405. [PMID: 1334406].
14. Peles E, Levy RB, Or E, Ullrich A, Yarden Y: **Oncogenic forms of the neu/HER2 tyrosine kinase are permanently coupled to phospholipase C gamma**. *The EMBO journal* 1991, **10**(8):2077–2086. [PMID: 1676673].
15. Sithanandam G, Anderson LM: **The ERBB3 receptor in cancer and cancer gene therapy**. *Cancer gene therapy* 2008, **15**(7):413–448. [PMID: 18404164].
16. Samuels Y, Diaz Jr LA, Schmidt-Kittler O, Cummins JM, DeLong L, Cheong I, Rago C, Huso DL, Lengauer C, Kinzler KW, Vogelstein B, Velculescu VE: **Mutant PIK3CA promotes cell growth and invasion of human cancer cells**. *Cancer Cell* 2005, **7**(6):561–573, [http://www.sciencedirect.com/science/article/pii/S1535610805001601].
17. Holbro T, Beerli RR, Maurer F, Koziczak M, Barbas r Carlos F, Hynes NE: **The ErbB2/ErbB3 heterodimer functions as an oncogenic unit: ErbB2 requires ErbB3 to drive breast tumor cell proliferation**. *Proceedings of the National Academy of Sciences of the United States of America* 2003, **100**(15):8933–8938. [PMID: 12853564].

18. An WL, Bjorkdahl C, Liu R, Cowburn RF, Winblad B, Pei JJ: **Mechanism of zinc-induced phosphorylation of p70 S6 kinase and glycogen synthase kinase 3beta in SH-SY5Y neuroblastoma cells**. *Journal of neurochemistry* 2005, **92**(5):1104–1115. [PMID: 15715661].

19. Dickler MN, Rugo HS, Eberle CA, Brogi E, Caravelli JF, Panageas KS, Boyd J, Yeh B, Lake DE, Dang CT, Gilewski TA, Bromberg JF, Seidman AD, D'Andrea GM, Moasser MM, Melisko M, Park JW, Dancey J, Norton L, Hudis CA: **A Phase II Trial of Erlotinib in Combination with Bevacizumab in Patients with Metastatic Breast Cancer**. *Clinical Cancer Research* 2008, **14**(23):7878–7883, [http://clincancerres.aacrjournals.org/content/14/23/7878].

20. Singer E, Landgraf R, Horan T, Slamon D, Eisenberg D: **Identification of a heregulin binding site in HER3 extracellular domain**. *The Journal of biological chemistry* 2001, **276**(47):44266–44274. [PMID: 11555649].

21. Butch ER, Guan KL: **Characterization of ERK1 activation site mutants and the effect on recognition by MEK1 and MEK2**. *The Journal of biological chemistry* 1996, **271**(8):4230–4235. [PMID: 8626767].

22. Hannan KM, Thomas G, Pearson RB: **Activation of S6K1 (p70 ribosomal protein S6 kinase 1) requires an initial calcium-dependent priming event involving formation of a high-molecular-mass signalling complex**. *The Biochemical journal* 2003, **370**(Pt 2):469–477. [PMID: 12429015].

23. Higuchi M, Onishi K, Kikuchi C, Gotoh Y: **Scaffolding function of PAK in the PDK1-Akt pathway**. *Nature cell biology* 2008, **10**(11):1356–1364. [PMID: 18931661].

24. Baselga J, Swain SM: **Novel anticancer targets: revisiting ERBB2 and discovering ERBB3**. *Nature reviews. Cancer* 2009, **9**(7):463–475. [PMID: 19536107].

25. Pandey GN, Dwivedi Y, SridharaRao J, Ren X, Janicak PG, Sharma R: **Protein kinase C and phospholipase C activity and expression of their specific isozymes is decreased and expression of MARCKS is increased in platelets of bipolar but not in unipolar patients**. *Neuropsychopharmacology: official publication of the American College of Neuropsychopharmacology* 2002, **26**(2):216–228. [PMID: 11790517].

26. B Dufourny vTH: **Stabilization of cyclin D1 mRNA via the phosphatidylinositol 3-kinase pathway in MCF-7 human breast cancer cells.** *The Journal of endocrinology* 2000, **166**(2):329–38.

27. Liang J, Slingerland JM: **Multiple roles of the PI3K/PKB (Akt) pathway in cell cycle progression**. *Cell cycle (Georgetown, Tex.)* 2003, **2**(4):339–345. [PMID: 12851486].

28. Rena G, Guo S, Cichy SC, Unterman TG, Cohen P: **Phosphorylation of the transcription factor forkhead family member FKHR by protein kinase B**. *The Journal of biological chemistry* 1999, **274**(24):17179–17183. [PMID: 10358075].

29. Zheng WH, Kar S, Quirion R: **Insulin-like growth factor-1-induced phosphorylation of the forkhead family transcription factor FKHRL1 is mediated by Akt kinase in PC12 cells**. *The Journal of biological chemistry* 2000, **275**(50):39152–39158. [PMID: 10995739].

30. Madrid LV, Mayo MW, Reuther JY, Baldwin J A S: **Akt stimulates the transactivation potential of the RelA/p65 Subunit of NF-kappa B through utilization of the Ikappa B kinase and activation of the mitogen-activated protein kinase p38**. *The Journal of biological chemistry* 2001, **276**(22):18934–18940. [PMID: 11259436].

31. Vander Haar E, Lee SI, Bandhakavi S, Griffin TJ, Kim DH: **Insulin signalling to mTOR mediated by the Akt/PKB substrate PRAS40**. *Nature cell biology* 2007, **9**(3):316–323. [PMID: 17277771].

32. Roux PP, Ballif BA, Anjum R, Gygi SP, Blenis J: **Tumor-promoting phorbol esters and activated Ras inactivate the tuberous sclerosis tumor suppressor complex via p90 ribosomal S6 kinase**. *Proceedings of the National Academy of Sciences of the United States of America* 2004, **101**(37):13489–13494. [PMID: 15342917].

33. Wulf GM, Ryo A, Wulf GG, Lee SW, Niu T, Petkova V, Lu KP: **Pin1 is overexpressed in breast cancer and cooperates with Ras signaling in increasing the transcriptional activity of c-Jun towards cyclin D1**. *The EMBO journal* 2001, **20**(13):3459–3472. [PMID: 11432833].

34. Luciani MG, Hutchins JR, Zheleva D, Hupp TR: **The C-terminal regulatory domain of p53 contains a functional docking site for cyclin A**. *Journal of molecular biology* 2000, **300**(3):503–518. [PMID: 10884347].

35. Mateyak MK, Obaya AJ, Sedivy JM: **c-Myc regulates cyclin D-Cdk4 and -Cdk6 activity but affects cell cycle progression at multiple independent points**. *Molecular and cellular biology* 1999, **19**(7):4672–4683. [PMID: 10373516].

36. Schulze WX, Deng L, Mann M: **Phosphotyrosine interactome of the ErbB-receptor kinase family**. *Molecular systems biology* 2005, **1**:2005.0008. [PMID: 16729043].

37. Creamer BA, Sakamoto K, Schmidt JW, Triplett AA, Moriggl R, Wagner KU: **Stat5 promotes survival of mammary epithelial cells through transcriptional activation of a distinct promoter in Akt1**. *Molecular and cellular biology* 2010, **30**(12):2957–2970. [PMID: 20385773].

38. Butt AJ, McNeil CM, Musgrove EA, Sutherland RL: **Downstream targets of growth factor and oestrogen signalling and endocrine resistance: the potential roles of c-Myc, cyclin D1 and cyclin E**. *Endocrine-related cancer* 2005, **12 Suppl 1**:S47–59. [PMID: 16113099].

39. Dougherty MK, Müller J, Ritt DA, Zhou M, Zhou XZ, Copeland TD, Conrads TP, Veenstra TD, Lu KP, Morrison DK: **Regulation of Raf-1 by direct feedback phosphorylation**. *Molecular cell* 2005, **17**(2):215–224. [PMID: 15664191].

40. Ravenhall C, Guida E, Harris T, Koutsoubos V, Stewart A: **The importance of ERK activity in the regulation of cyclin D1 levels and DNA synthesis in human cultured airway smooth muscle**. *British Journal of Pharmacology* 2000, **131**:17–28, [http://www.ncbi.nlm.nih.gov/pmc/articles/PMC1572283/]. [PMID: 10960064 PMCID: PMC1572283].

41. Ma L, Chen Z, Erdjument-Bromage H, Tempst P, Pandolfi PP: **Phosphorylation and functional inactivation of TSC2 by Erk implications for tuberous sclerosis and cancer pathogenesis**. *Cell* 2005, **121**(2):179–193. [PMID: 15851026].

42. Wang H, Garcia CA, Rehani K, Cekic C, Alard P, Kinane DF, Mitchell T, Martin M: **IFN-beta production by TLR4-stimulated innate immune cells is negatively regulated by GSK3-beta**. *Journal of immunology (Baltimore, Md.: 1950)* 2008, **181**(10):6797–6802. [PMID: 18981097].

43. Al-Khouri AM, Ma Y, Togo SH, Williams S, Mustelin T: **Cooperative phosphorylation of the tumor suppressor phosphatase and tensin homologue (PTEN) by casein kinases and glycogen synthase kinase 3beta**. *The Journal of biological chemistry* 2005, **280**(42):35195–35202. [PMID: 16107342].

44. Han JM, Park BJ, Park SG, Oh YS, Choi SJ, Lee SW, Hwang SK, Chang SH, Cho MH, Kim S: **AIMP2/p38, the scaffold for the multi-tRNA synthetase complex, responds to genotoxic stresses via p53**. *Proceedings of the National Academy of Sciences of the United States of America* 2008, **105**(32):11206–11211. [PMID: 18695251].

45. Ababneh M, Götz C, Montenarh M: **Downregulation of the cdc2/Cyclin B Protein Kinase Activity by Binding of p53 to p34cdc2**. *Biochemical and Biophysical Research Communications* 2001, **283**(2):507–512, [http://www.sciencedirect.com/science/article/pii/S0006291X01947928].

46. Watcharasit P, Bijur GN, Zmijewski JW, Song L, Zmijewska A, Chen X, Johnson GVW, Jope RS: **Direct, activating interaction between glycogen synthase kinase-3beta and p53 after DNA damage**. *Proceedings of the National Academy of Sciences of the United States of America* 2002, **99**(12):7951–7955. [PMID: 12048243].

47. Wu Y, Dowbenko D, Spencer S, Laura R, Lee J, Gu Q, Lasky LA: **Interaction of the tumor suppressor PTEN/MMAC with a PDZ domain of MAGI3, a novel membrane-associated guanylate kinase**. *The Journal of biological chemistry* 2000, **275**(28):21477–21485. [PMID: 10748157].

48. Freeman DJ, Li AG, Wei G, Li HH, Kertesz N, Lesche R, Whale AD, Martinez-Diaz H, Rozengurt N, Cardiff RD, Liu X, Wu H: **PTEN tumor suppressor regulates p53 protein levels and activity through phosphatase-dependent and -independent mechanisms**. *Cancer cell* 2003, **3**(2):117–130. [PMID: 12620407].

49. Qiu W, Wu J, Walsh EM, Zhang Y, Chen CY, Fujita J, Xiao ZXJ: **Retinoblastoma protein modulates gankyrin-MDM2 in regulation of p53 stability and chemosensitivity in cancer cells**. *Oncogene* 2008, **27**(29):4034–4043. [PMID: 18332869].

50. Inoki K, Li Y, Xu T, Guan KL: **Rheb GTPase is a direct target of TSC2 GAP activity and regulates mTOR signaling**. *Genes & development* 2003, **17**(15):1829–1834. [PMID: 12869586].
