# Supplementary figures and images for: Boolean ErbB network reconstructions and perturbation simulations reveal individual drug response in different breast cancer cell lines
Source: BMC Syst Biol. 2014 Jun 25;8:75. doi: 10.1186/1752-0509-8-75 (PMC4087127; doi:10.1186/1752-0509-8-75)

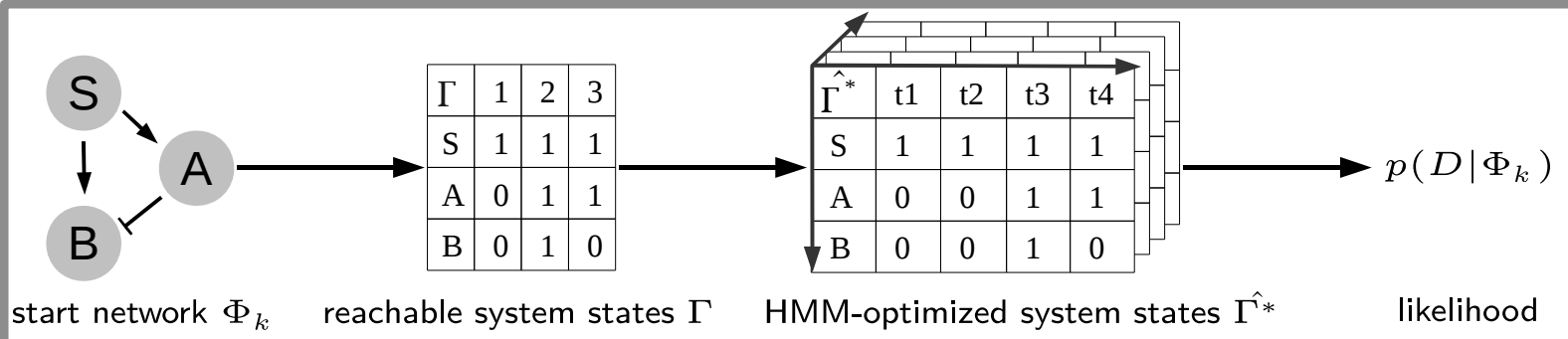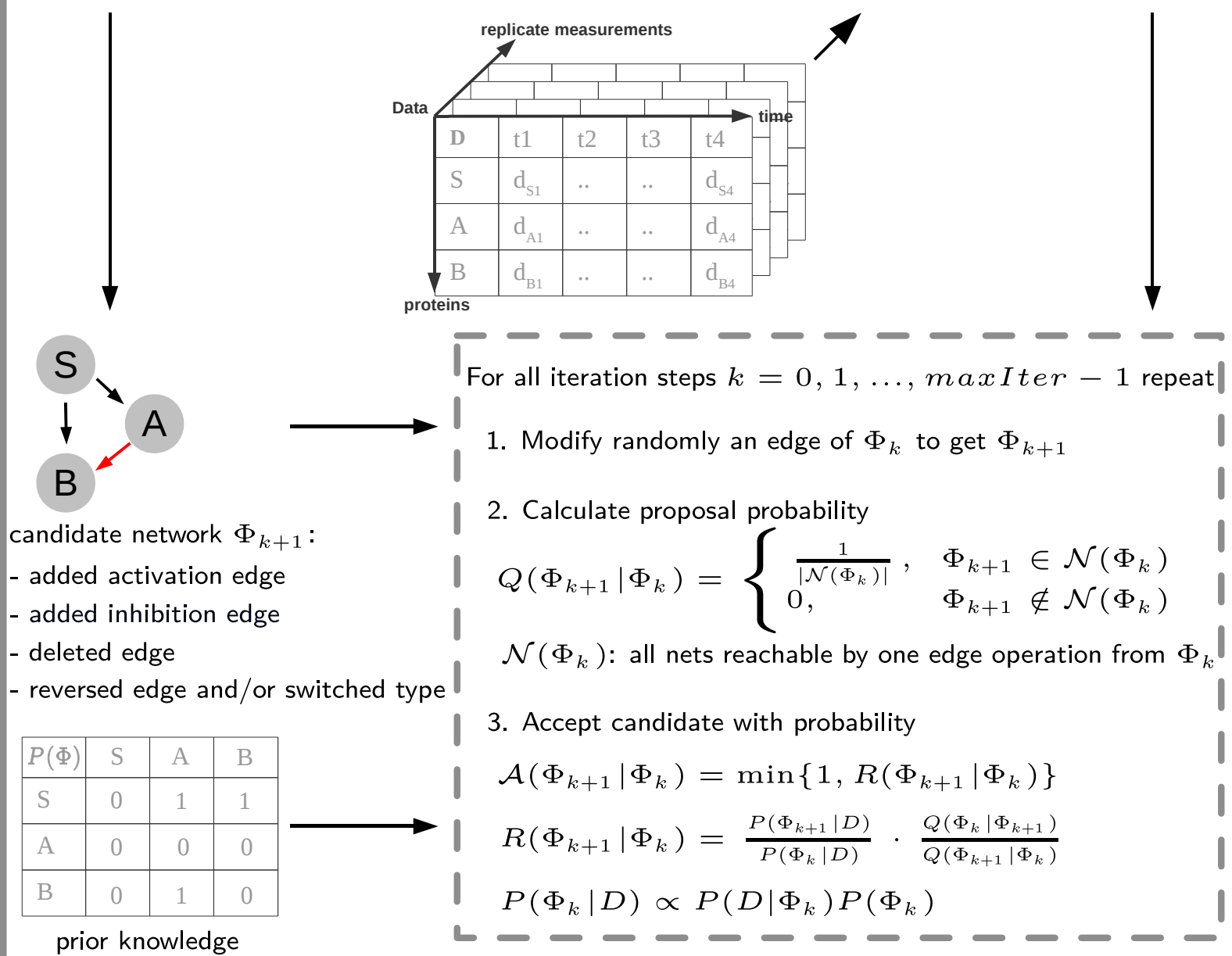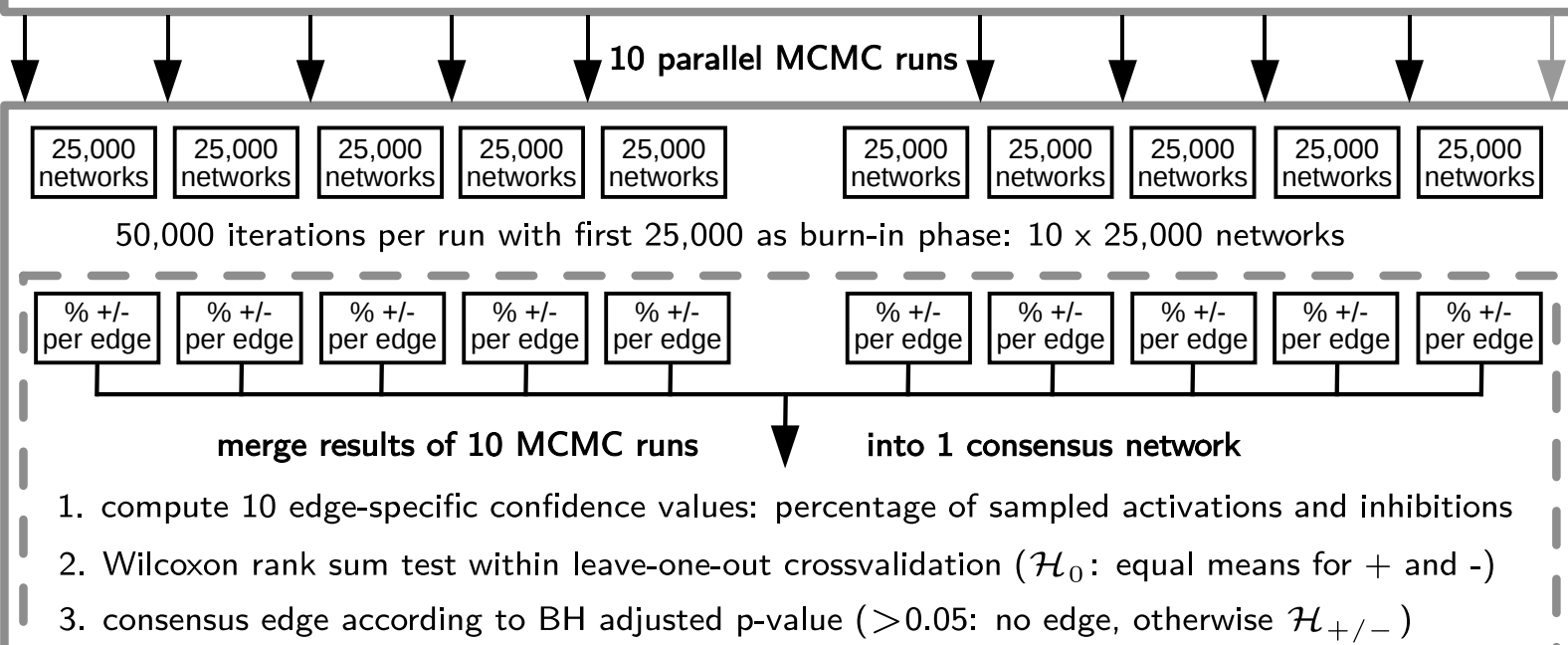

Supplement: Additional file 3 — Workflow of MCMC-based network structure inference. The inhibMCMC procedure of the ddepn package was run with maxIter = 50,000 in 10 parallel runs. The results of the 25,000 iterations after the burn-in phase were merged into one consensus network. It was applied for short- and long-term data separately per cell line, leading to six consensus networks. The figure is based on [6,47]. [file 1752-0509-8-75-S3.pdf]

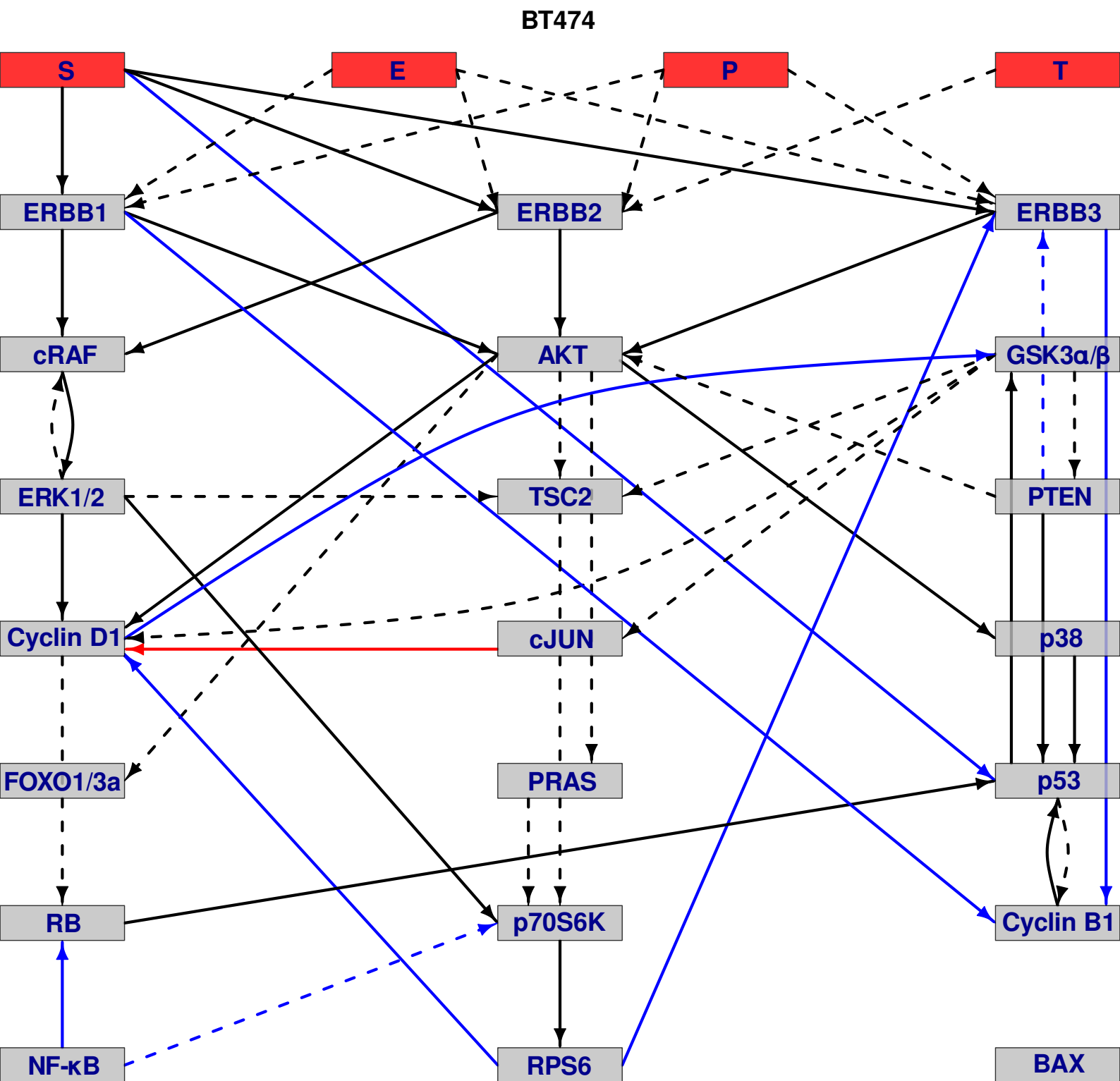

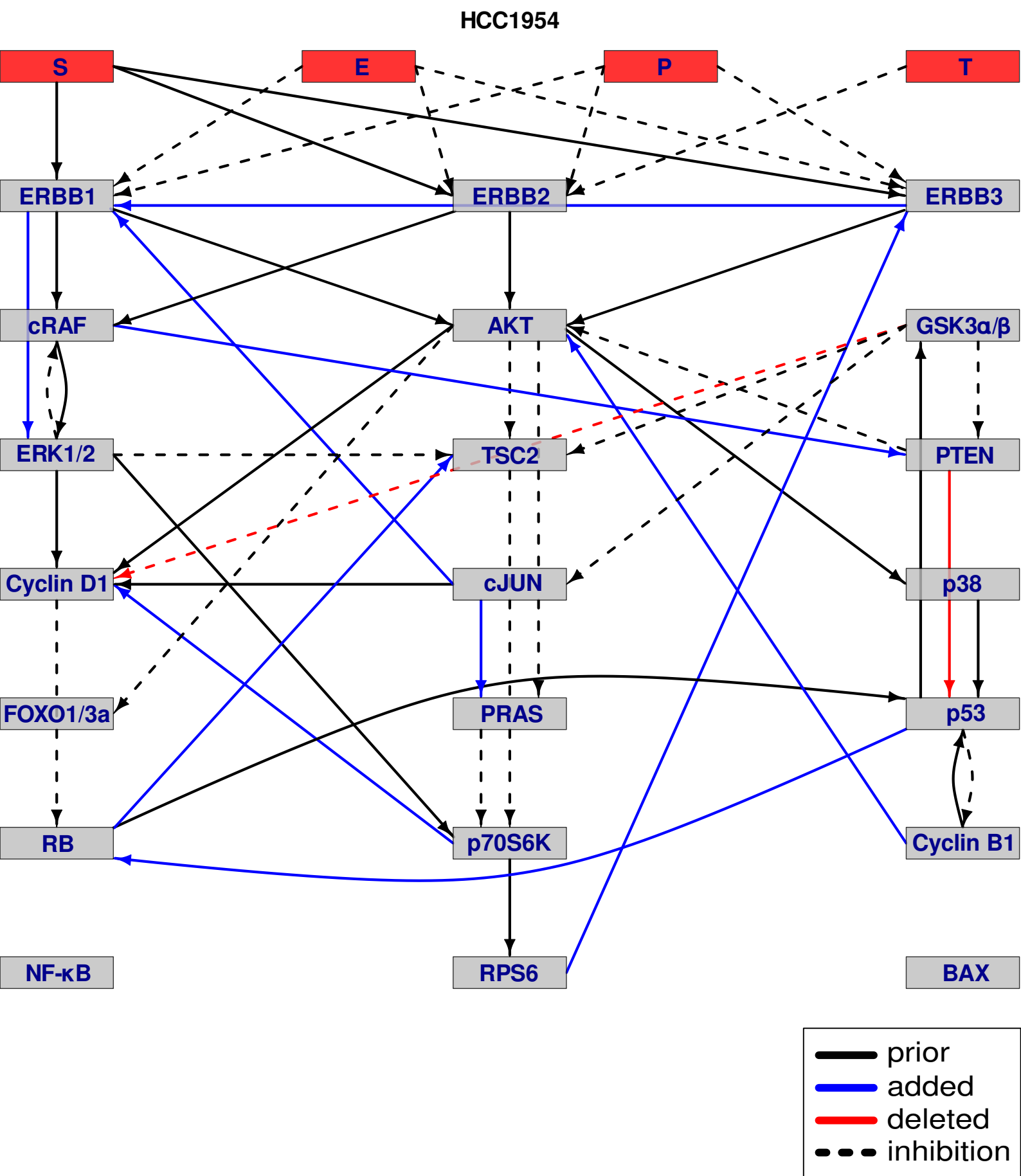

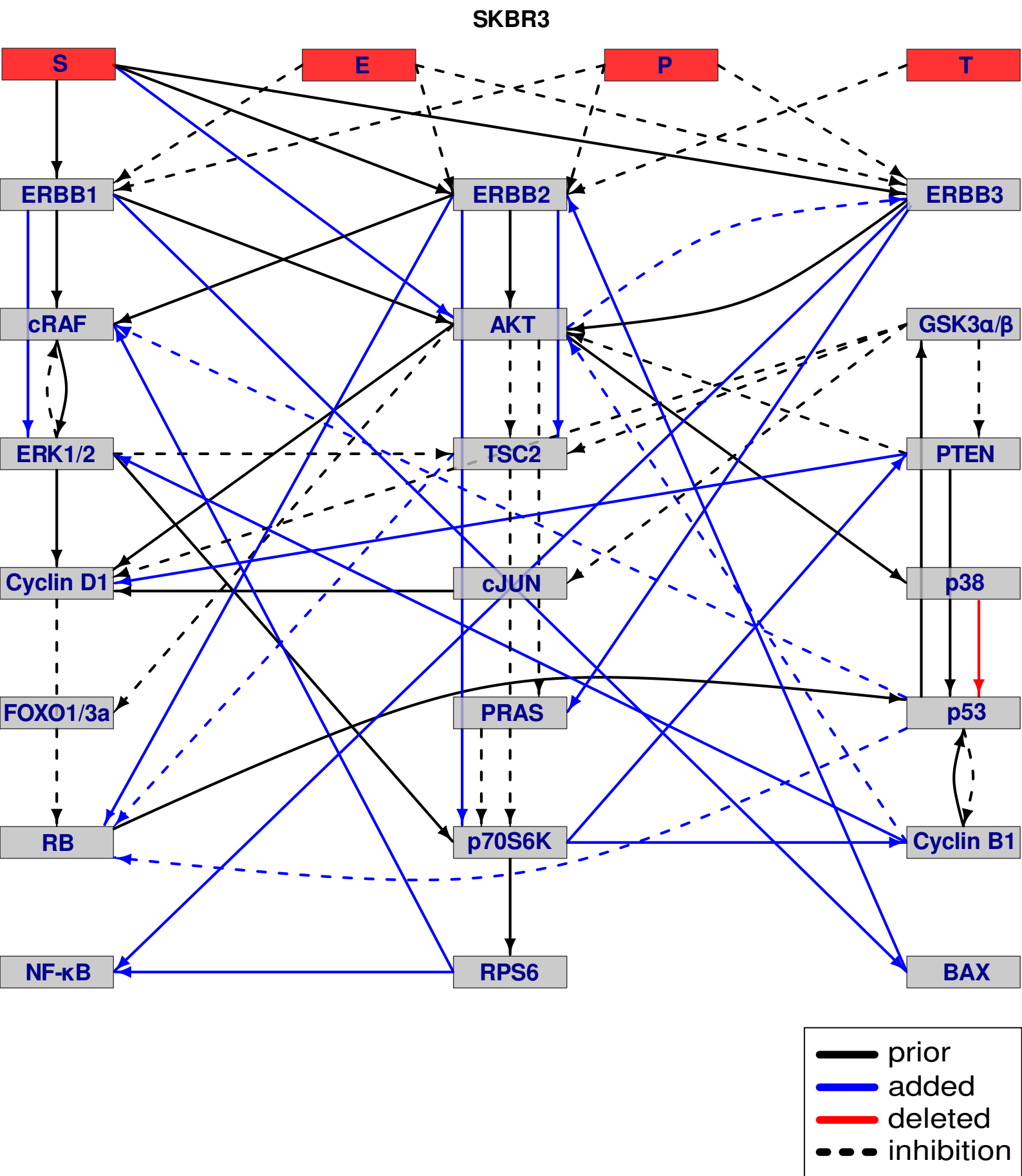

Supplement: Additional file 6 — Reconstructed long-term signalling networks. The figure displays the reconstructed long-term signalling networks for BT474, HCC1954 and SKBR3. Target proteins are represented as rectangles with stimulus and drugs coloured in red. The three drug names erlotinib, trastuzumab and pertuzumab are abbreviated via their first letters. Stimulation via full growth medium is denoted by S. Solid arrows denote activating interactions while dashed ones represent inhibitions. [file 1752-0509-8-75-S6.pdf]
