# Supplementary material for: Boolean ErbB network reconstructions and perturbation simulations reveal individual drug response in different breast cancer cell lines
Source: BMC Syst Biol. 2014 Jun 25;8:75. doi: 10.1186/1752-0509-8-75 (PMC4087127; doi:10.1186/1752-0509-8-75)
Supplement: Additional file 4 — Edge confidences for the reconstructed networks of the three cell lines per time course. In each of the ten MCMC runs, activation and inhibition edges were sampled. The percentage, i.e. the confidence, of sampled activation (red) and inhibition (blue) edges in the 25,000 iterations after the burn-in phase are depicted in the boxes. The sink nodes are displayed in each panel, while the activating, inhibiting or missing influence of the source nodes is shown column-wise in the red, blue or missing boxes. The source node names are displayed at the x-axis with additional indicators, where ‘-’ refers to an inhibiting influence and ‘+’ is related to activation. The x-axis of the short-term plots is labelled as ‘AKT, E, EGF, ERBB1, ERBB2, ERBB3, ERK1/2, HRG, MEK1/2, mTOR, P, p70S6K, PDK1, PKC α, PLC γ, T’. The x-axis of the long-term plots is labelled as ‘AKT, BAX, cJUN, cRAF, CyclinB1, CyclinD1, E, ERBB1, ERBB2, ERBB3, ERK1/2, FOXO1/3a, GSK3 α/ β, NF- κB, P, p38, p53, p70S6K, PRAS, PTEN, RB, S, RPS6, T, TSC2’. An activating edge in the consensus network, as described in Additional file 3, means that the sampled activating edges have a significantly higher confidence value than the inhibiting ones. As self-loops and ingoing edges to the drug or growth factor nodes were not allowed during inference, the respective confidences are zero. [file 1752-0509-8-75-S4.pdf]

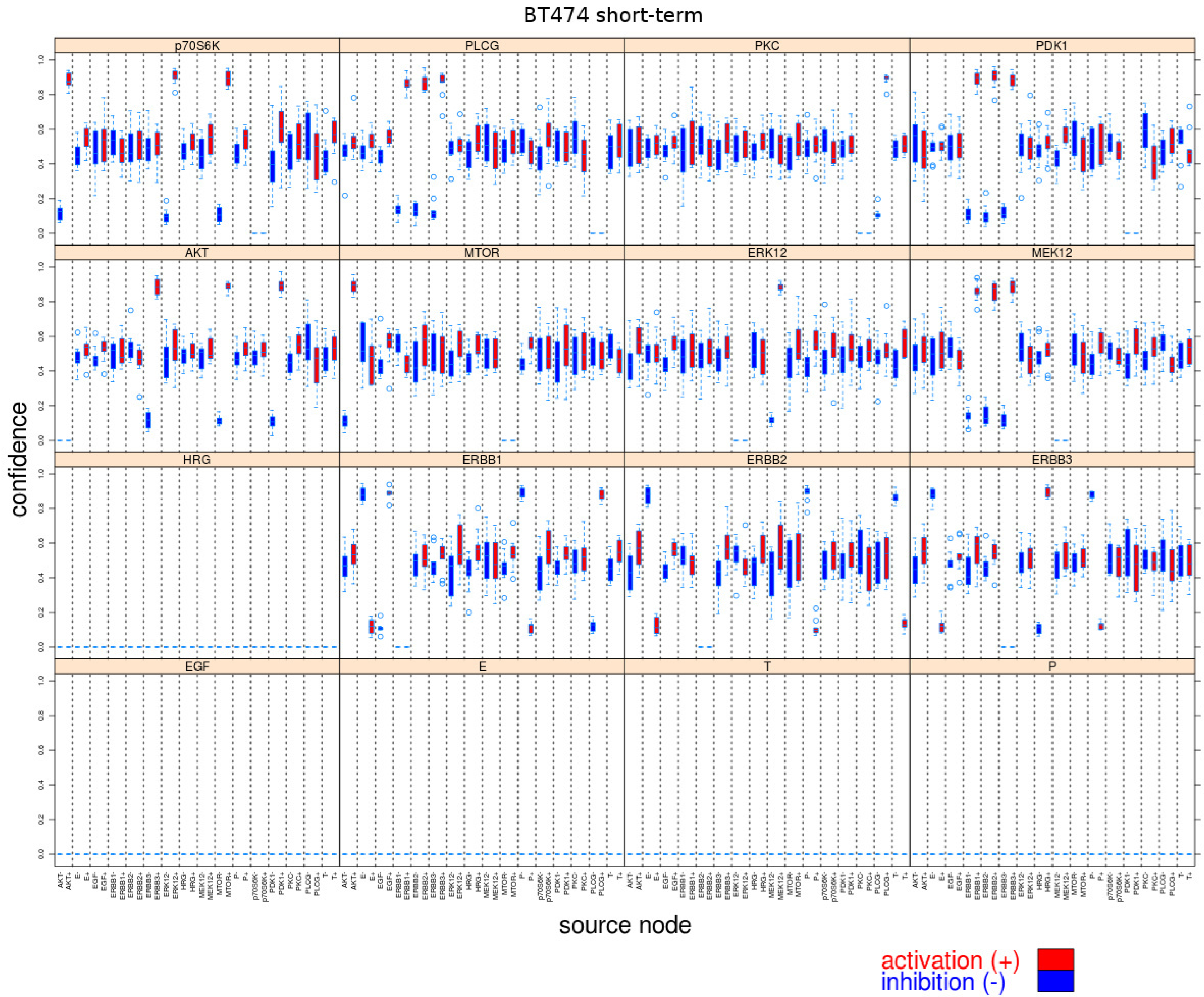

BT474 short-term
p70S6K
PLCG
PKC
PDK1
AKT
MTOR
ERK12
MEK12
HRG
ERBB1
ERBB2
ERBB3
EGF
E
T
P
confidence
source node
activation (+)
inhibition (-)

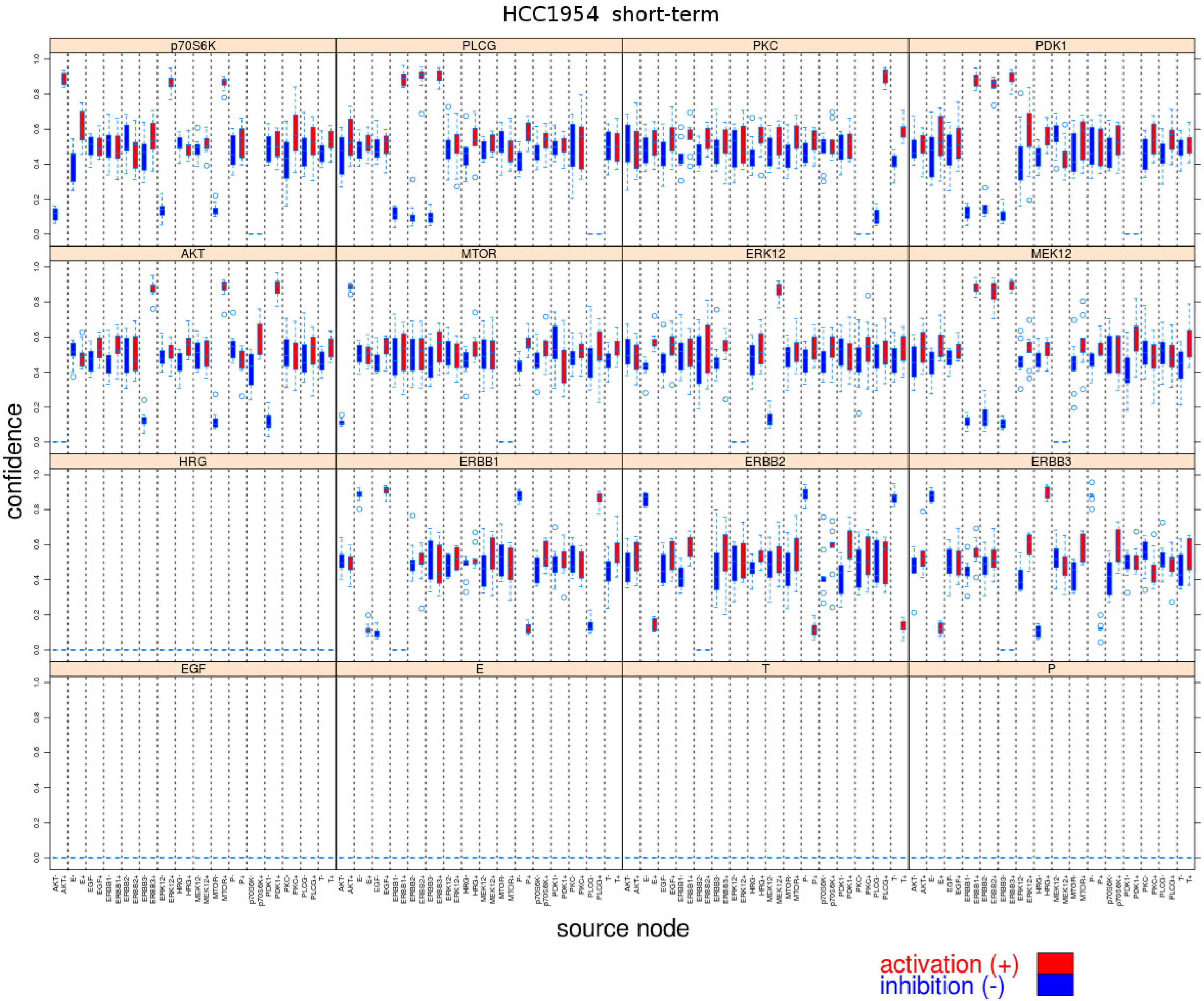

HCC1954 short-term
p70S6K
PLCG
PKC
PDK1
AKT
MTOR
ERK12
MEK12
HRG
ERBB1
ERBB2
ERBB3
EGF
E
T
P
confidence
source node
activation (+)
inhibition (-)

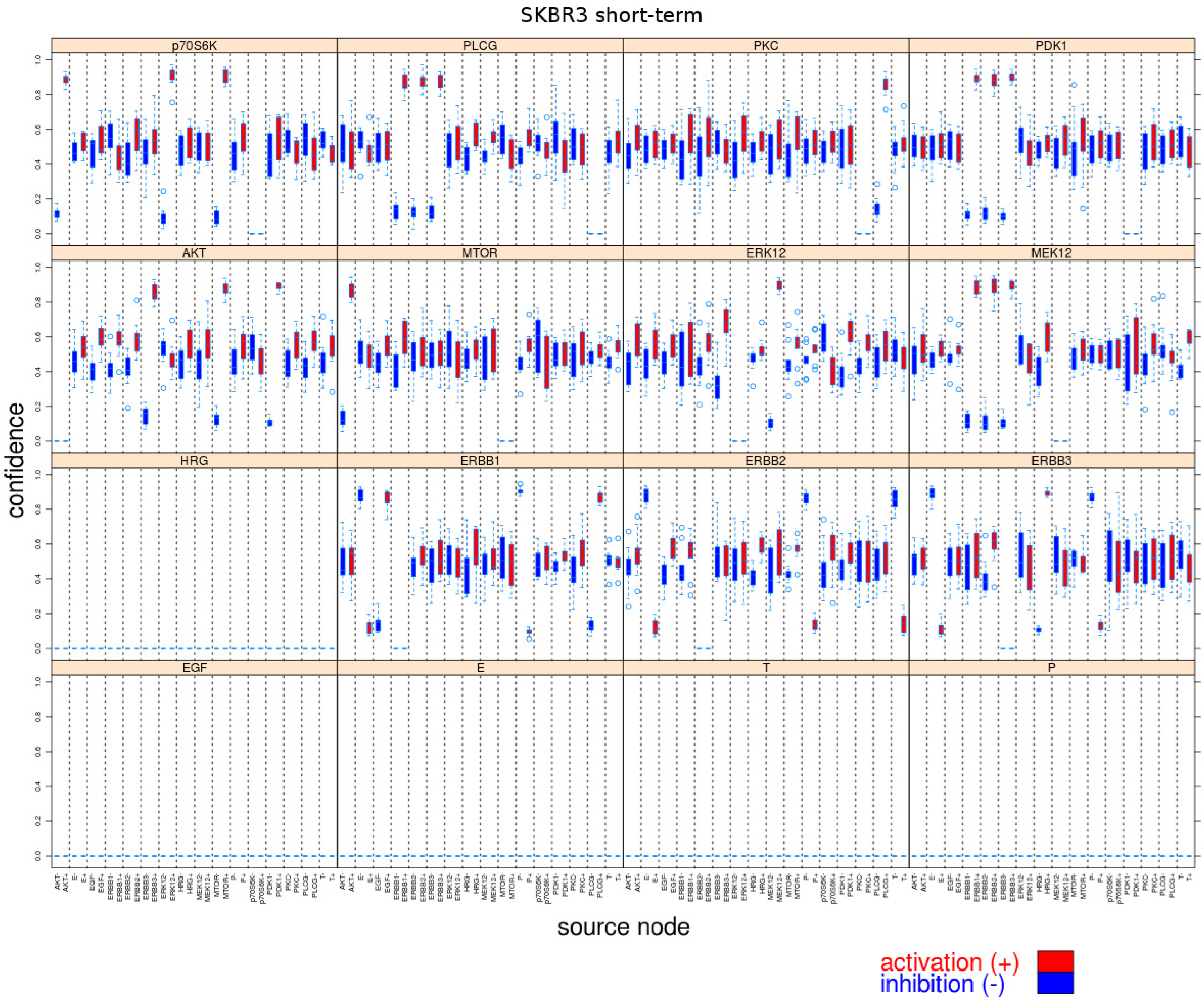

SKBR3 short-term
p70S6K
PLCG
PKC
PDK1
AKT
MTOR
ERK12
MEK12
HRG
ERBB1
ERBB2
ERBB3
EGF
E
T
P
confidence
source node
activation (+)
inhibition (-)

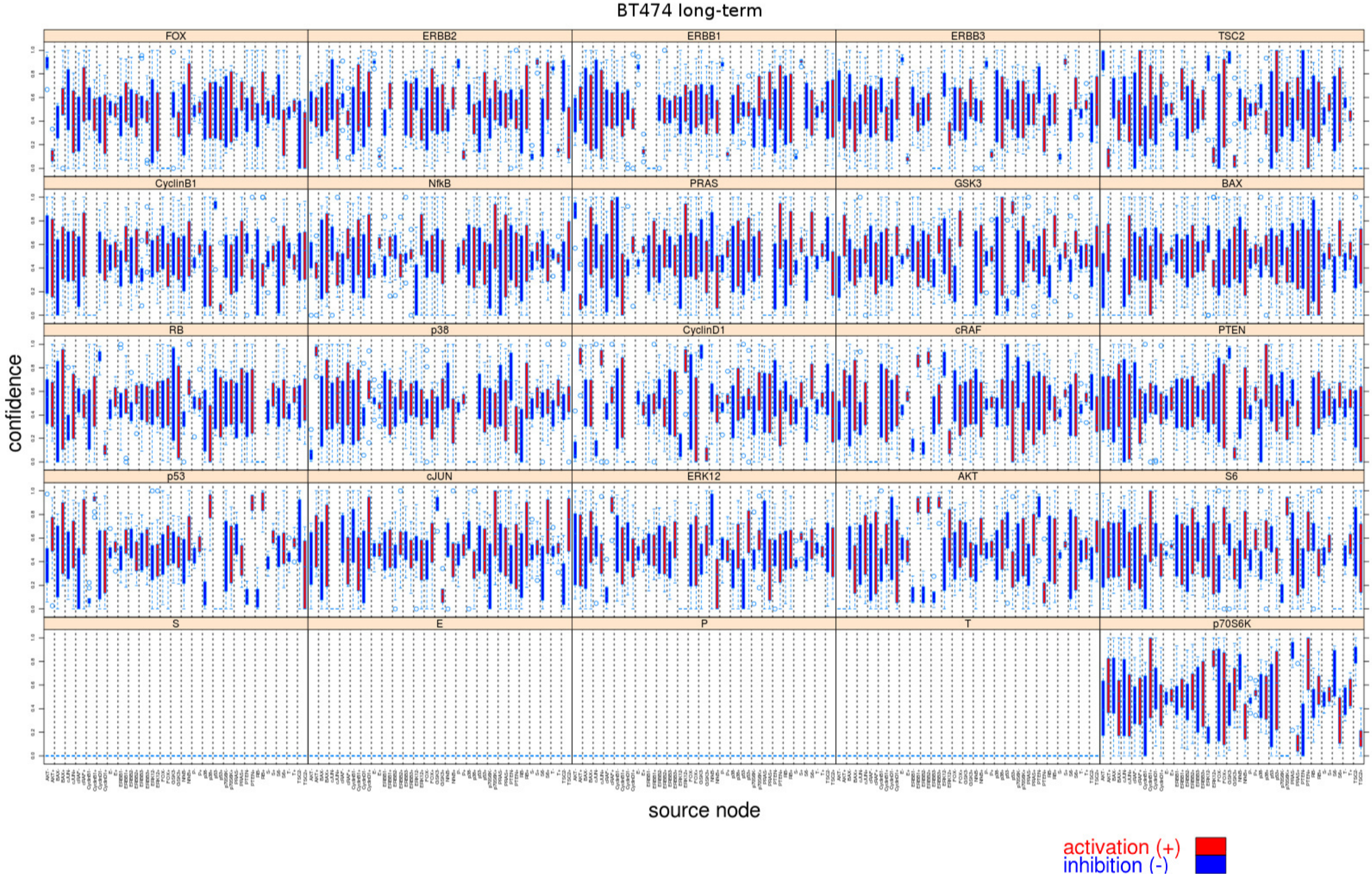

BT474 long-term
FOX
ERBB2
ERBB1
ERBB3
TSC2
CyclinB1
NfkB
PRAS
GSK3
BAX
RB
p38
CyclinD1
cRAF
PTEN
p53
cJUN
ERK12
AKT
S6
S
E
P
T
p70S6K
confidence
source node
activation (+)
inhibition (-)

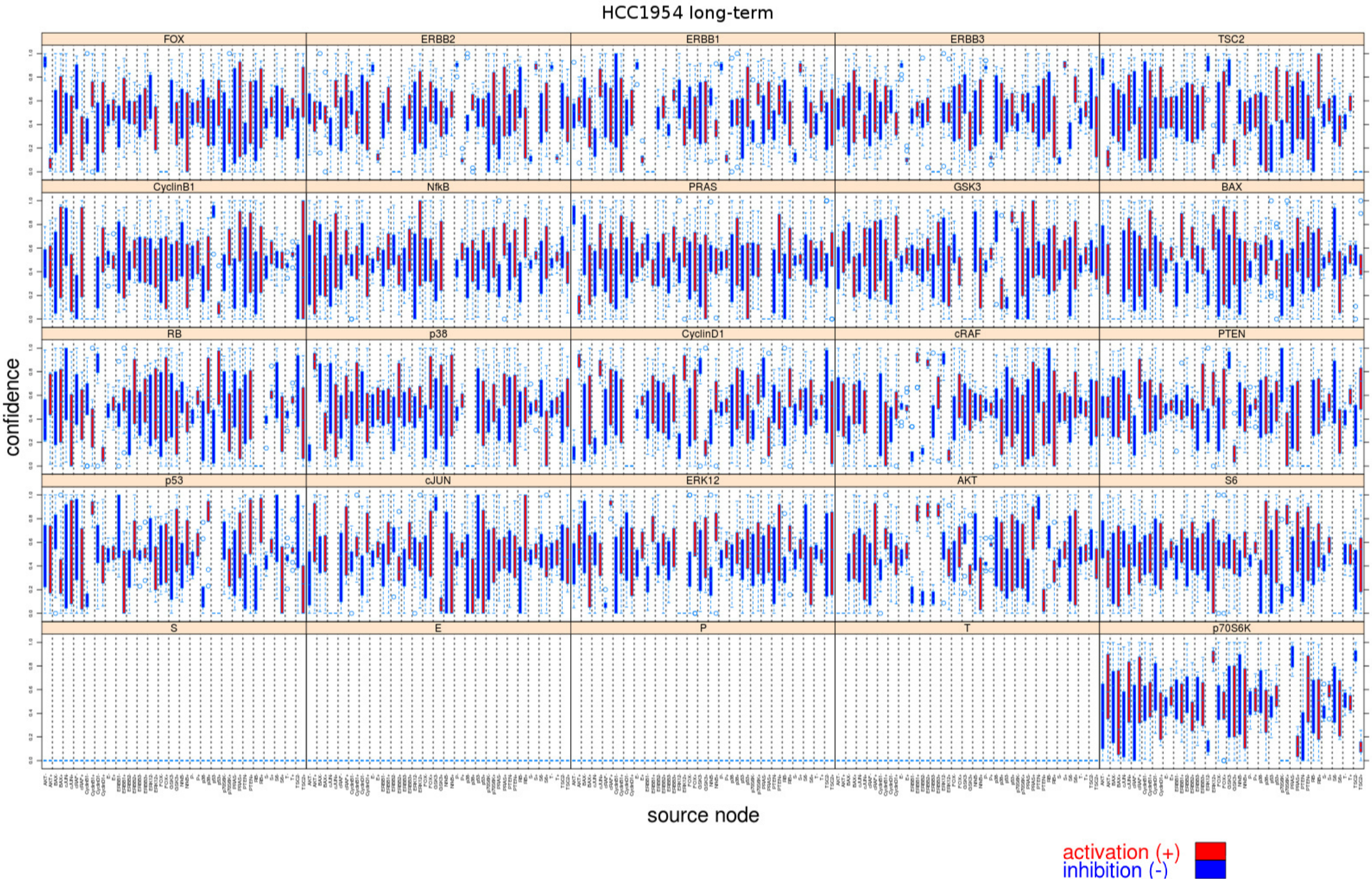

HCC1954 long-term
FOX
ERBB2
ERBB1
ERBB3
TSC2
CyclinB1
NfkB
PRAS
GSK3
BAX
RB
p38
CyclinD1
cRAF
PTEN
p53
cJUN
ERK12
AKT
S6
S
E
P
T
p70S6K
confidence
source node
activation (+)
inhibition (-)

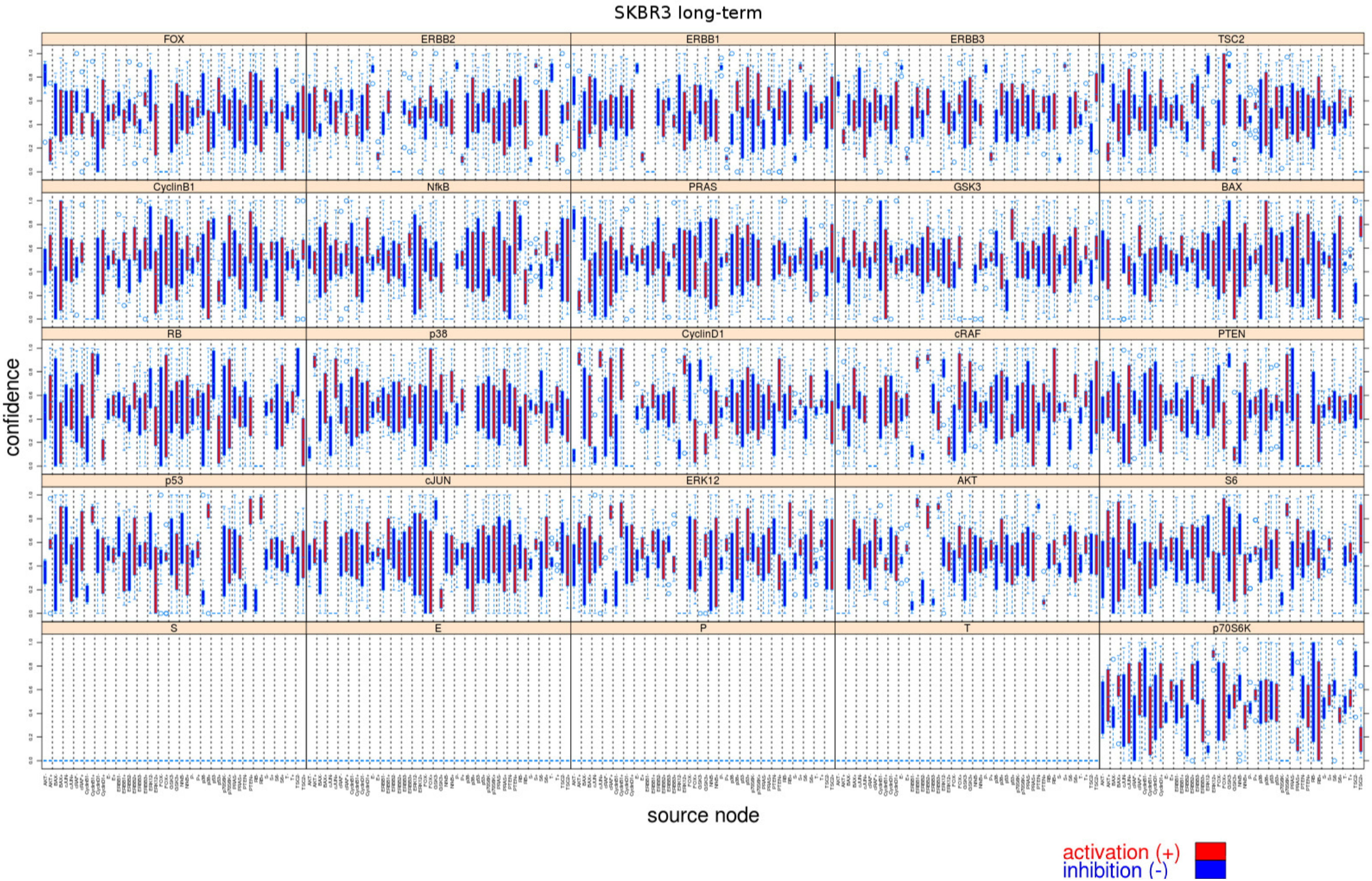
SKBR3 long-term
FOX
ERBB2
ERBB1
ERBB3
TSC2
CyclinB1
NfkB
PRAS
GSK3
BAX
RB
p38
CyclinD1
cRAF
PTEN
p53
cJUN
ERK12
AKT
S6
S
E
P
T
p70S6K
confidence
source node
activation (+)
inhibition (-)
