# Supplementary material for: Boolean ErbB network reconstructions and perturbation simulations reveal individual drug response in different breast cancer cell lines
Source: BMC Syst Biol. 2014 Jun 25;8:75. doi: 10.1186/1752-0509-8-75 (PMC4087127; doi:10.1186/1752-0509-8-75)
Supplement: Additional file 5 — Boolean interaction rules for the components of the short- and long-term signalling networks. The tables contain the rules that arose from network reconstructions based on short- and long-term RPPA data of BT474, HCC1954 and SKBR3. The three drug names erlotinib, trastuzumab and pertuzumab are abbreviated via their first letters. For the long-term networks, the stimulus is denoted by S. Symbols are interpretable in the following way: & ≡ AND, ∨≡ OR and ! ≡ NOT. [file 1752-0509-8-75-S5.pdf]

Additional file 5 — Boolean interaction rules for the components of the short- and long-term signalling networks

The tables contain the rules that arose from network reconstructions based on short- and long-term RPPA data of BT474, HCC1954 and SKBR3. The three drug names erlotinib, trastuzumab and pertuzumab are abbreviated via their first letters. For the long-term networks, the stimulus is denoted by S. Symbols are interpretable in the following way: & ≡ AND, ∨ ≡ OR and ! ≡ NOT.

**Short-term network for BT474**	
Target protein	Activation rules
AKT	AKT ∨ ERBB3 ∨ mTOR ∨ PDK1
ERBB1	(ERBB1 ∨ EGF ∨ PLCγ) & !(E ∨ P)
ERBB2	(ERBB2 ∨ EGF ∨ ERBB3) & !(E ∨ T ∨ P)
ERBB3	(ERBB3 ∨ HRG) & !(E ∨ P)
ERK1/2	ERK1/2 ∨ EGF ∨ P ∨ MEK1/2
MEK1/2	MEK1/2 ∨ ERBB1 ∨ ERBB2 ∨ ERBB3
mTOR	mTOR ∨ P ∨ AKT
p70S6K	p70S6K ∨ AKT ∨ mTOR ∨ ERK1/2
PDK1	PDK1 ∨ ERBB1 ∨ ERBB2 ∨ ERBB3 ∨ MEK1/2
PKCα	PKCα ∨ PLCγ
PLCγ	PLCγ ∨ EGF ∨ ERBB1 ∨ ERBB2 ∨ ERBB3

**Short-term network for HCC1954**	
Target protein	Activation rules
AKT	AKT ∨ ERBB3 ∨ mTOR ∨ p70S6K ∨ PDK1
ERBB1	(ERBB1 ∨ EGF ∨ PLCγ) & !(E ∨ P)
ERBB2	(ERBB2 ∨ ERBB1 ∨ PDK1) & !(E ∨ T ∨ P)
ERBB3	(ERBB3 ∨ HRG ∨ ERK1/2) & !(E ∨ P)
ERK1/2	ERK1/2 ∨ MEK1/2
MEK1/2	MEK1/2 ∨ ERBB1 ∨ ERBB2 ∨ ERBB3 ∨ PDK1
mTOR	mTOR ∨ AKT
p70S6K	p70S6K ∨ E ∨ AKT ∨ mTOR ∨ ERK1/2
PDK1	PDK1 ∨ ERBB1 ∨ ERBB2 ∨ ERBB3
PKCα	PKCα ∨ T ∨ PLCγ
PLCγ	PLCγ ∨ ERBB1 ∨ ERBB2 ∨ ERBB3

Short-term network for SKBR3	
Target protein	Activation rules
AKT	AKT $\vee$ ERBB3 $\vee$ mTOR $\vee$ PDK1
ERBB1	(ERBB1 $\vee$ EGF $\vee$ PLCγ) & !(E $\vee$ P)
ERBB2	ERBB2 & !(E $\vee$ T $\vee$ P)
ERBB3	(ERBB3 $\vee$ HRG) & !(E $\vee$ P)
ERK1/2	ERK1/2 $\vee$ ERBB3 $\vee$ MEK1/2 $\vee$ PDK1
MEK1/2	MEK1/2 $\vee$ ERBB1 $\vee$ ERBB2 $\vee$ ERBB3
mTOR	mTOR $\vee$ AKT
p70S6K	p70S6K $\vee$ AKT $\vee$ mTOR $\vee$ ERK1/2
PDK1	PDK1 $\vee$ ERBB1 $\vee$ ERBB2 $\vee$ ERBB3
PKCα	PKCα $\vee$ PLCγ
PLCγ	PLCγ $\vee$ ERBB1 $\vee$ ERBB2 $\vee$ ERBB3

Long-term network for BT474	
Target protein	Activation rules
AKT	(AKT $\vee$ ERBB2 $\vee$ ERBB1 $\vee$ ERBB3) & !PTEN
BAX	BAX
cJUN	cJUN & !GSK3α/β
cRAF	(cRAF $\vee$ ERBB2 $\vee$ ERBB1) & !ERK1/2
Cyclin B1	(Cyclin B1 $\vee$ ERBB1 $\vee$ ERBB3) & !p53
Cyclin D1	(Cyclin D1 $\vee$ ERK1/2 $\vee$ AKT $\vee$ RPS6) & !GSK3α/β
ERBB1	(ERBB1 $\vee$ S) & !(E $\vee$ P)
ERBB2	(ERBB2 $\vee$ S) & !(E $\vee$ P $\vee$ T)
ERBB3	(ERBB3 $\vee$ S $\vee$ RPS6) & !(E $\vee$ P $\vee$ PTEN)
ERK1/2	ERK1/2 $\vee$ cRAF
FoxO1/3a	FoxO1/3a & !AKT
GSK3α/β	GSK3α/β $\vee$ p53 $\vee$ Cyclin D1
NF-κB	NF-κB
p38	p38 $\vee$ AKT
p53	p53 $\vee$ S $\vee$ RB $\vee$ p38 $\vee$ PTEN $\vee$ Cyclin B1
p70S6K	(p70S6K $\vee$ ERK1/2) & !(NF-κB $\vee$ PRAS $\vee$ TSC2)
PRAS	PRAS & !AKT
PTEN	PTEN & !GSK3α/β
RB	(RB $\vee$ NF-κB) & !Cyclin D1
RPS6	RPS6 $\vee$ p70S6K
TSC2	TSC2 & !(ERK1/2 $\vee$ AKT $\vee$ GSK3α/β)

Long-term network for HCC1954	
Target protein	Activation rules
AKT	(AKT $\vee$ Cyclin B1 $\vee$ ERBB2 $\vee$ ERBB1 $\vee$ ERBB3) & !PTEN
BAX	BAX
cJUN	cJUN & !GSK3α/β
cRAF	(cRAF $\vee$ ERBB2 $\vee$ ERBB1) & !ERK1/2
Cyclin B1	Cyclin B1 & !p53
Cyclin D1	Cyclin D1 $\vee$ p70S6K $\vee$ cJUN $\vee$ ERK1/2 $\vee$ AKT
ERBB1	(ERBB1 $\vee$ S $\vee$ cJUN $\vee$ ERBB3) & !(E $\vee$ P)
ERBB2	(ERBB2 $\vee$ S) & !(E $\vee$ P $\vee$ T)
ERBB3	(ERBB3 $\vee$ S $\vee$ RPS6) & !(E $\vee$ P)
ERK1/2	ERK1/2 $\vee$ cRAF $\vee$ ERBB1
FoxO1/3a	FoxO1/3a & !AKT
GSK3α/β	GSK3α/β $\vee$ p53
NF-κB	NF-κB
p38	p38 $\vee$ AKT
p53	p53 $\vee$ RB $\vee$ p38 $\vee$ Cyclin B1
p70S6K	(p70S6K $\vee$ ERK1/2) & !(PRAS $\vee$ TSC2)
PRAS	(PRAS $\vee$ cJUN) & !AKT
PTEN	(PTEN $\vee$ cRAF) & !GSK3α/β
RB	(RB $\vee$ p53) & !Cyclin D1
RPS6	RPS6 $\vee$ p70S6K
TSC2	(TSC2 $\vee$ RB) & !(ERK1/2 $\vee$ AKT $\vee$ GSK3α/β)

Long-term network for SKBR3	
Target protein	Activation rules
AKT	(AKT $\vee$ S $\vee$ ERBB2 $\vee$ ERBB1 $\vee$ ERBB3) & !(PTEN $\vee$ Cyclin B1)
BAX	BAX $\vee$ ERBB1
cJUN	cJUN & !GSK3α/β
cRAF	(cRAF $\vee$ RPS6 $\vee$ ERBB2 $\vee$ ERBB1) & !(p53 $\vee$ ERK1/2)
Cyclin B1	(Cyclin B1 $\vee$ p70S6K) & !p53
Cyclin D1	(Cyclin D1 $\vee$ cJUN $\vee$ ERK1/2 $\vee$ AKT $\vee$ PTEN) & !GSK3α/β
ERBB1	(ERBB1 $\vee$ S) & !(E $\vee$ P)
ERBB2	(ERBB2 $\vee$ S $\vee$ BAX) & !(E $\vee$ P $\vee$ T)
ERBB3	(ERBB3 $\vee$ S) & !(E $\vee$ P $\vee$ AKT)
ERK1/2	ERK1/2 $\vee$ cRAF $\vee$ Cyclin B1 $\vee$ ERBB1
FoxO1/3a	FoxO1/3a & !AKT
GSK3α/β	GSK3α/β $\vee$ p53
NF-κB	NF-κB $\vee$ RPS6 $\vee$ ERBB3
p38	p38 $\vee$ AKT
p53	p53 $\vee$ RB $\vee$ PTEN $\vee$ Cyclin B1
p70S6K	(p70S6K $\vee$ ERK1/2 $\vee$ ERBB2) & !(PRAS $\vee$ TSC2)
PRAS	(PRAS $\vee$ ERBB3) & !AKT
PTEN	(PTEN $\vee$ p70S6K) & !GSK3α/β
RB	(RB $\vee$ ERBB2) & !(p53 $\vee$ Cyclin D1 $\vee$ TSC2)
RPS6	RPS6 $\vee$ p70S6K
TSC2	(TSC2 $\vee$ ERBB2) & !(ERK1/2 $\vee$ AKT $\vee$ GSK3α/β)
